# A fertility-restoring genotype of beet (*Beta vulgaris* L.) is composed of a weak *restorer-of-fertility* gene and a modifier gene tightly linked to the *Rf1* locus

**DOI:** 10.1371/journal.pone.0198409

**Published:** 2018-06-01

**Authors:** Takumi Arakawa, Daisuke Uchiyama, Takashi Ohgami, Ryo Ohgami, Tomoki Murata, Yujiro Honma, Hiroyuki Hamada, Yosuke Kuroda, Kazunori Taguchi, Kazuyoshi Kitazaki, Tomohiko Kubo

**Affiliations:** 1 Research Faculty of Agriculture, Hokkaido University, Sapporo, Japan; 2 Hokkaido Agricultural Research Center, National Agriculture and Food Research Organization, Memuro, Japan; New Mexico State University, UNITED STATES

## Abstract

Cytoplasmic male sterility (CMS) is a plant trait that involves interactions between nuclear- and mitochondrial genomes. In CMS, the nuclear *restorer-of-fertility* gene (*Rf*), a suppressor of male-sterility inducing mitochondria, is one of the best known genetic factors. Other unidentified genetic factors may exist but have not been well characterized. In sugar beet (*Beta vulgaris* L.), CMS is used for hybrid seed production, but few male-sterility inducing nuclear genotypes exist. Such genotypes could be introduced from a closely related plant such as leaf beet, but first the fertility restoring genotype of the related plant must be characterized. Here, we report the discovery of a Japanese leaf beet accession ‘Fukkoku-ouba’ that has both male-sterility inducing and fertility restoring genotypes. We crossed the leaf beet accession with a sugar beet CMS line, developed succeeding generations, and examined the segregation of two DNA markers that are linked to two sugar beet *Rf*s, *Rf1* and *Rf2*. Only the *Rf2* marker co-segregated with fertility restoration in every generation, implying that the *Rf1* locus in leaf beet is occupied by a non-restoring allele. Fertility restoration was incomplete without a genetic factor closely linked to *Rf1*, leading to the assumption that the *Rf1* locus encodes a modifier that cannot restore fertility by itself but perhaps strengthens another *Rf*. We sequenced the apparently non-restoring ‘Fukkoku-ouba’ *rf1* gene-coding region and found that it closely resembles a restoring allele. The protein product demonstrated its potential to suppress CMS in transgenic suspension cells. In contrast, ‘Fukkoku-ouba’ *rf1* transcript abundance was highly reduced compared to that of the restoring *Rf1*. Consistently, changes in protein complexes containing CMS-associated mitochondrial protein in anthers were very minor. Accordingly, we concluded that ‘Fukkoku-ouba’ *rf1* is a hypomorph that acts as a non-restoring allele but has the potential to support another *Rf*, i.e. it is a modifier candidate.

## Introduction

Mitochondria are endosymbiotic organelles that originated from α-proteobacteria [1]. Although mitochondria possess their own genomes, their genetic information is insufficient to sustain mitochondrial function, for which a large number of nuclear genes are required [2]. Improper mitochondrial-nuclear interaction can be manifested in such processes as lifespan, female gametogenesis or male gametogenesis [3] [4] [5]. The effect of mitochondrial-nuclear interaction on male gametogenesis has been well studied in plants and is known as cytoplasmic male sterility (CMS) [6].

CMS has been found in more than 150 species [7]. Expression of CMS is explained by a genetic model in which mitochondrial factor S, ‘sterile’, that causes male sterility interacts with nuclear factor *Rf*, ‘restorer of fertility’, whose dominant allele suppresses the action of S. In this genetic model, male sterility is expressed only when the S plant has a non-restoring allele of *Rf*, i.e. the genotype [S]*rfrf*.

There may be one or more *Rf* genes present in the genome [8]. For example, CMS-T, one of the three CMS types in maize, requires *Rf1* (or another equivalent gene) and *Rf2* for fertility restoration [9]. In addition, a modifier gene that does not restore pollen fertility by itself but modifies the action of *Rf* was proposed when segregation of pollen fertility was complex. Duvick [10] suggested the involvement of a modifier gene in fertility restoration in some crossing experiments associated with maize CMS-T. Thus, some minor genes are perhaps involved in CMS expression, implying that CMS is under the control of an elaborate genetic system.

CMS is an important character in agriculture because it provides ideal seed parents for large-scale hybrid seed production [11]. Sugar beet [*Beta vulgaris* ssp. *vulgaris* (Sugar Beet Group)] is representative of crops in which CMS is used to develop commercial varieties. Sugar beet breeders use a single source of CMS that was discovered by Owen [12]. Because CMS plants cannot self-pollinate, their propagation requires a specific pollen parental line that has the same nuclear genotype as the CMS line (i.e. being devoid of any dominant *Rf*), but the pollen parental line is male fertile due to normal mitochondria. Such pollen parental lines are called maintainers. Because visual discrimination of an *Rf* plant from an *rf* plant is impossible, selection of maintainers requires a test cross; a plant is a maintainer genotype if all the F1 between the plant and the CMS plant are male sterile. In sugar beet, the frequency of the maintainer genotype is generally less than 5% [13], making the non-restoring genotype invaluable. Identification of non-restoring genotypes is one of the major tasks in sugar beet breeding.

To explain the expression of sugar beet CMS, Owen posited a genetic model composed of S and two *Rf* genes, *X* and *Z*, of which the former is the stronger restorer [12]. Later, the two *Rf*s were assigned to chromosomes 3 and 4, respectively [14] [15]. Owen’s model is now generally accepted, but some researchers have observed such a complex segregation of fertility restoration in their genetic analyses that additional genetic factors have been incorporated into their genetic models to explain their observations. For example, Hogaboam [16] postulated a modifier gene, *Sh* (shrunken anthers), that strengthens the effect of *Rf*. Details about *Sh* are unknown. In his genetic model, *Rf* was linked to a gene controlling the number of embryos in a seed (designated as *M* after monogerm). At present, the *M* locus is assigned to chromosome 4 [15]. Therefore, the *Rf* mentioned in Hogaboam’s report is likely *Z*, although he assumed it was *X* [16], implying an epistasis between *Sh* and *Z*. The underlying mechanism in complex fertility restoration could be disentangled by molecular study.

The molecular biology of plant CMS has been studied for decades. With a few exceptions [17] [18], the molecular entity of S is thought to be a unique ORF that is composed of gene-coding, gene-flanking, and/or origin-unknown sequences [19]. Such ORFs, hereafter termed *S-orf*, have been reported in many plant species [20]. The lack of conservation in the nucleotide sequences of these *S-orf*s suggests that their origins are evolutionarily independent. Protein products of the *S-orf*s have been detected either specifically in anthers [21] [22] or from all the plant organs examined e. g. [23].

Molecular effects of *Rf* on mitochondria leads to diverse events or processes such as elimination of the DNA segment containing *S-orf* [24] and aldehyde metabolism [25]. The most intensively studied cases are associated with a post-transcriptional mechanism in *S-orf* expression that results in the decrease of S-ORF protein products [6]. *Rf*s responsible for the post-transcriptional mechanism encode pentatricopeptide repeat (PPR) proteins [26]. Unlike PPR-associated fertility restoration, studies on other classes of fertility restoration are relatively rare.

In sugar beet, *S-orf* is an N-terminal extension of *atp6* that encodes a hydrophobic protein of 387 amino acid residues [27]. This *S-orf*, termed *preSatp6*, is constitutively expressed, and its protein products form homo-oligomers [27] similar to the S-*orf* in other plants, such as maize *urf13-T* and radish *orf138* [28] [29]. Of the two sugar beet *Rf*s, *X* was cloned as *Rf1* [30]. The *Rf1* locus contains a gene cluster consisting of genes resembling *Oma1*, a gene known to be involved in the quality control of mitochondrial inner membrane proteins and mitochondrial dynamics [31]. One of the clustered genes, termed *orf20*, has the strongest ability to restore fertility in transgenic sugar beets compared with the other clustered genes [30]. Expression of *orf20* is most prominent in young anthers, and concomitantly, the homo-oligomer form of preSATP6 protein is reduced but the amount of preSATP6 protein is almost unchanged, suggesting alteration in the higher order structure of the preSATP6 protein [32]. The protein complex that specifically occurs in fertility-restored anthers is also present in transgenic suspension cells expressing *orf20* [32]. This specific complex contains both ORF20 and preSATP6 proteins [32]. Because ORF20 protein binds with preSATP6 protein, ORF20 protein appears to play like a molecular chaperone [32]. On the other hand, although *Rf2* was genetically identified as a counterpart of *Z* [33], *Rf2* remains uncharacterized.

Organizational comparison at the molecular level of the *Rf1* locus in various sugar beets revealed multiple variants whose diversity is unknown [34]. For the sake of maintainer identification, *rf1* organization in maintainers was investigated [34] [35]. The results showed that three molecular variants (and two derivatives) have been selected as non-restoring alleles by sugar beet breeders, of which one variant is predominant. This finding raises a concern about the genetic vulnerability caused by repeated selection of a single allele [36]. Because sugar beet was produced from a specific type of fodder beet by mass selection [37], the lack of molecular diversity in the non-restoring allele can be a reflection of the small founder population. In this regard, characterization of the maintainer genotype in the other *B*. *vulgaris* groups (e.g. leaf beet, garden beet, and fodder beet) should be interesting. Identification of maintainer genotypes could be facilitated by the knowledge of fertility restoring genotypes in these beet groups.

Here, we report the genetic analysis of a leaf beet accession from which a fertility-restoring genotype and a maintainer genotype were found. The fertility-restoring genotype was dissected into two genetic factors: a weak *Rf* that is tightly linked (or allelic) to *Rf2* and a modifier gene that is tightly linked to *Rf1*. From the maintainer genotype, a novel *rf1* molecular variant was found. From our analysis, this *rf1* variant appeared to be fixed in this leaf beet accession. Interestingly, our molecular analysis indicated the hypomorphic nature of this *rf1* variant. Although the *rf1* variant has the potential to alter the higher order structure of the preSATP6 protein, the amount of its mRNA is reduced compared to the restoring allele. We propose a model suggesting that this *rf1* variant acts as a modifier and discuss the implications of modifier genes on sugar beet breeding.

## Materials and methods

### Plant materials

Seeds of leaf beet [*B*. *vulgaris* ssp. *vulgaris* (Leaf Beet Group)] accession ‘Fukkoku-ouba’, an old Japanese landrace, were obtained from Genebank, National Agriculture and Food Research Organization (NARO), Japan (accession number JP25745). Sugar beet line TA-33BB-CMS is a CMS line and TA-33BB-O is its maintainer inbred line [35]. Sugar beet line NK-198 has S mitochondria but is male fertile due to *Rf1* [30]. NK-219mm-CMS is another CMS sugar beet inbred line that is compatible with transgenic experiments [38]. All the sugar beet lines were developed by Hokkaido Agricultural Research Center, NARO. Plants were grown in the field of the Field Science Center for Northern Biosphere, Hokkaido University, or in the environment-controlled greenhouse of Research Faculty of Agriculture, Hokkaido University (kept in the range of 20–25°C and illuminated by incandescent light at night). For crossing experiments, inflorescences were paper-bag enclosed before anthesis. After anthesis, inflorescences of pollen-parents were placed in the bags [35].

### Evaluation of male fertility

Male fertility is classified into four classes based on anther morphology and color. A fully fertile anther is apparently indistinguishable from the maintainer line and produces as much pollen as the maintainer line does. Semi-fertile (type a) anthers produce pollen but the amount is less than for the fully fertile, and the anther color is more yellowish. Semi-fertile (type b) anthers rarely dehisce and their color is orangish. Completely sterile anthers are shriveled, and their color is white or brown. Plants with fully fertile and type-a semi-fertile anthers were combined into the normal class in some genetic analyses. For male-fertility indexing, each plant was evaluated as described above and scored more than four times on different days as follows: a score of 3 for fully fertile, 2 for type-a semi-fertile, 1 for type-b semi-fertile, and 0 for completely sterile, respectively. The average of the scores was the plant’s fertility index.

### Genotyping by DNA markers

Total cellular DNA of green leaves was isolated according to the method of Doyle and Doyle [39]. DNA markers used in this study are summarized in Table 1. Details of o7 will be described elsewhere, but tight linkage between o7 and ca4 (an *rf2*-linked DNA marker reported by Honma et al. [33]) is shown in S1 Text. PCR products were electrophoresed in 0.8 or 2% agarose gels, depending on the DNA fragment size.

**Table 1:** Summary of DNA markers used in this study.

Name	Type^1^	Nucleotide sequences of PCR primers	RE^2^	Reference
s17	CAPS	5'-CAATCTGTGGTGCTGACCAAA-3'	*Hin*dIII and *Msp*I	[36]
		5'-GATTAAAGAGGGCTGCTGAAGCCGAGA-3'		
o7	DFLP	5'-CTAAGAAATACTTCATCCCATGTCCTGC-3'	-	This study
		5'-TGACCAAGATCCCAAGATTTGATATGG -3'		

^1^CAPS, cleaved amplified polymorphic sequence; DFLP, DNA fragment length polymorphism.

^2^Restriction endonucleases for polymorphism detection.

### Statistical and quantitative trait loci analyses

Statistical tests were done at the website (http://aoki2.si.gunma-u.ac.jp/exact/exact.html; accessed on September 30, 2017) or using Microsoft Excel (Microsoft Japan, Tokyo, Japan).

### DNA gel blot analysis

Total cellular DNA of green leaves was purified by cesium-chloride continuous density gradients [40] and digested with *Hin*dIII (Takara Bio, Kusatsu, Japan). DNA fragments were electrophoresed in 1.0% agarose gels and transferred to Hybond N+ membranes (GE Healthcare, Little Chalfont, UK) according to the instruction manual. Preparation and labeling probe DNA followed the procedure described in Ohgami et al. [35].

### Determination and analysis of nucleotide sequences

The nucleotide sequence of *orf20*_*fukkoku*_ was determined by an ABI3130 sequence analyzer (Thermo Fisher Scientific, Waltham, MA, USA) as described in Ohgami et al. [35]. Sequence data were deposited in DDBJ/EMBL/GenBank under the accession number LC325860. Analysis of nucleotide sequences was done by the Sequencher program (Hitachi Solutions, Tokyo, Japan), the BLAST program (https://blast.ncbi.nlm.nih.gov/Blast.cgi), and ClustalW (http://clustalw.ddbj.nig.ac.jp/index.php?lang=ja).

### Production of transgenic suspension cells

The binary vector containing *orf20*_*fukkoku*_::*flag* was constructed as follows: the *orf20*_*fukkoku*_ ORF and its 5'- and 3'-UTRs were PCR amplified with a pair of primers (5'-GGGGACAAGTTTGTACAAAAAAGCAGGCTAGGAATATCATAACCATT-3' and 5'-GGGGACCACTTTGTACAAGAAAGCTGGGTTGCATGGGGTAATCACATCCA-3') from genomic DNA of ‘Fukkoku-ouba’. The resulting PCR fragment was inserted into pDONRzeo (Invitrogen, Carlsbad, CA, USA) using the BP Clonase Enzyme mix (Invitrogen) according to the manufacturer’s instructions. To add a *flag* tag, plasmid DNA was PCR amplified with two overlapping primers (5'-TCAGGATTATAAGGATGATGATGATAAGTGACCATTTACCAACCAGCATCTTCTTTTAGCAGCTT-3' and 5'-GTCACTTATCATCATCATCCTTATAATCCTGAAGACCTTGAATTGCACGTCCTGCTACAA-3'). The two primers were designed to fuse the *flag* tag to the 3'-end of *bvORF20*_*fukkoku*_ and are partially complementary to each other. The PCR products were digested with *Dpn*I to destroy residual plasmid DNA and then introduced into *E*. *coli* by electroporation. Circular plasmid DNA was restored in transformed *E*. *coli* via DNA recombination. After confirming the nucleotide sequence of the recovered plasmid DNA from a colony grown on a selective plate, the insert DNA was transferred to pMDC [32] using the LR Clonase Enzyme mix (Invitrogen). The resultant binary vector *orf20*_*fukkoku*_::*flag* was introduced into sugar beet suspension cells. Two constructs, *orf20*_*NK-198*_::*flag* and *orf20L*::*flag* [32], were used as controls. The procedure for transformation was as described in Kagami *et al*. [41].

### Blue-Native polyacrylamide gel electrophoresis (BN-PAGE)

Crude mitochondria were isolated from transgenic calli according to the method of Kitazaki *et al*. [32]. Proteins from immature anthers and crude mitochondria for BN-PAGE were prepared according to the method of Kitazaki *et al*. [32]. BN-PAGE was performed using the Native PAGE Novex BisTris Gel system (Invitrogen), according to the manufacturer’s instructions.

### Immunoblot analysis

Protein samples were boiled in 2 x SDS-PAGE buffer [4% (w/v) SDS, 20% (v/v) glycerol, 2% (v/v) 2-mercaptoethanol, 0.002% (w/v) bromophenol blue, 100 mM Tris-HCl, pH 6.8] for 5 min, then subjected to SDS-PAGE according to the method of Schägger and von Jagow [42]. Separated proteins from SDS-PAGE and BN-PAGE were subjected to immunoblot analysis according to the method of Kitazaki *et al*. [32]. Anti-FLAG antibody (αFLAG) was purchased from MBL (http://www.mbl.co.jp) and was diluted to 50 ng/mL when used for immunoblot analysis. αpreSATP6 [27] was diluted to 42.5 ng/mL or 0.34 μg/mL for SDS-PAGE and BN-PAGE, respectively. αCOXI [27] was diluted to 42.5 ng/mL.

### Quantitative reverse transcription PCR

Collected anthers were sorted according to their developmental stages. Total cellular RNA from anthers was extracted with an RNeasy Plant Mini Kit (Qiagen, Valencia, CA, USA) and treated with RNase-free DNase I (Takara Bio). RNA samples (300 ng) were reverse-transcribed with SuperScript III First-Strand Synthesis System (Invitrogen) using an oligo dT primer. The resultant cDNA was mixed with primers (0.2 μM each) and PowerUp SYBR Green Master Mix (Life Technologies, Carlsbad, CA, USA). Nucleotide sequences of the primers were: *orf20*-like genes, 5'-GGAAGAAGCATAGTGGGGCT-3' and 5'-CACAGCATGCCCAACCTGAT-3'; *elongation factor 1α* (*ef1α*), 5'-TGAGGCTGGTATCTCCAAGG-3' and 5'-TTGAGTACTTGGGGGTGGTG-3'; and *Actin*, 5'-AGACCTTCAATGTGCCTGCT-3' and 5'-ACGACCAGCAAGATCCAAAC-3'. PCR was monitored with a Chromo 4, Opticon Monitor (ver. 3.1) with a DNA-Engine PTC-200 (Bio-Rad Laboratories, Hercules, CA, USA). The PCR protocol was 50 °C for 2 min, 95 °C for 2 min, and then 40 cycles of 95 °C for 15 s and 60.8 °C for 1 min. After the quantification, all the reactants were heated to 95°C (1 min) and then cooled to 50°C. A melting-curve was then drawn (53 to 87°C, data acquisition every 0.5°C) to verify that there was a single amplicon. The delta-Ct method was used for quantification of Ct values [43].

## Results

### Maintainer and fertility-restoring genotypes in a Japanese leaf beet accession

In our attempt to identify maintainer genotypes in beets, we selected two Japanese leaf beets (accession ‘Fukkoku-ouba’) and crossed them with a sugar beet CMS line, TA-33BB-CMS. Male fertility of the F1 plants is shown in Table 2. All the F1 plants using pollen parent #1 were completely sterile, whereas normal (pollen-shedding) and completely sterile plants segregated in the F1 when pollen parent #2 was used. Therefore, ‘Fukkoku-ouba’ appeared to be an accession containing both maintainer and fertility-restoring genotypes.

**Table 2:** Male fertility of F1 plants in two test crosses of TA-33BB-CMS x ‘Fukkoku-ouba’.

Pollen parental plant	Male fertility^1^			Total
	N	Sf	CS	
#1	0	0	11	11
#2	7	0	7	14

^1^N, normal; Sf, semi-fertile; and CS, completely sterile.

### A *Restorer-of-fertility* gene in ‘Fukkoku-ouba’

**Fig 1:**
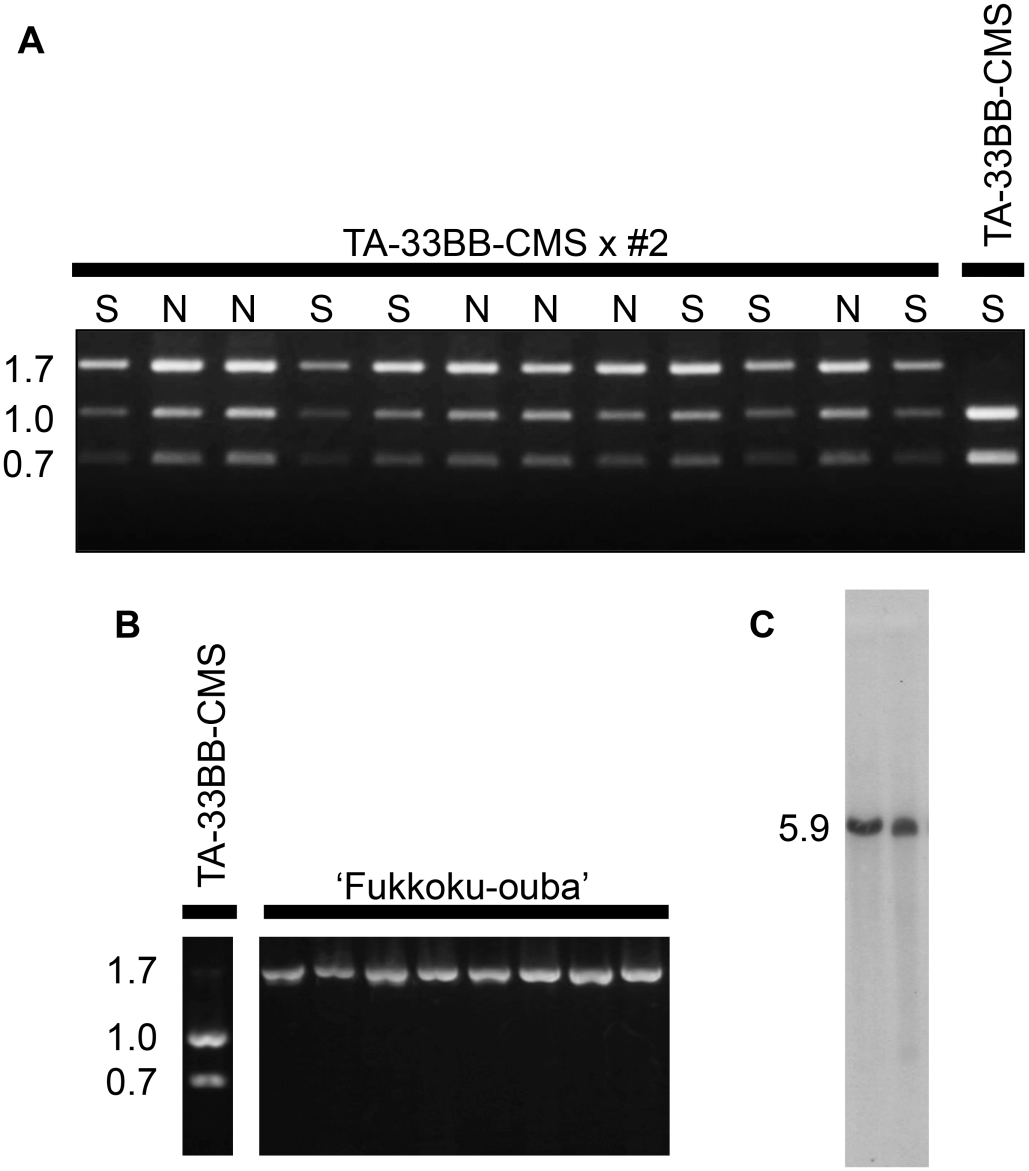
Marker analysis of s17 in F1 plants and a ‘Fukkoku-ouba’ DNA gel blot probed with the *orf20*-3' UTR. (A) Agarose gel electrophoresis of s17 cleaved amplified fragments derived from representative F1 plants of the cross TA-33BB-CMS x ‘Fukkoku-ouba’ #2 and a TA-33BB-CMS plant. Male fertility of each plant is indicated as S, completely sterile, or N, normal. Size markers are shown on the left (kbp). (B) Agarose gel electrophoresis of s17 markers derived from TA-33BB-CMS and ‘Fukkoku-ouba’ plants. Size markers are shown on the left (kbp). (C) A DNA gel blot containing two genomic DNAs of ‘Fukkoku-ouba’ plants probed with the *orf20*-3' UTR. Size markers are shown on the left (kbp).

To determine which of the two *Rf*s were associated with the fertility-restoring genotype of leaf beet, we first examined the allele type of the *Rf1* locus in F1 plants. We used the s17 marker, a cleaved amplified polymorphic sequence marker (co-dominant) that is located ~4 kbp downstream of the *orf20*-like gene cluster [36]. In our previous study, we found five electrophoretic patterns for s17, and named them patterns 1 to 5 [36]. For example, TA-33BB-CMS is homozygous for pattern 4 and yields an electrophoretic pattern with 1.0-, and 0.7-kbp bands [35]. All the 25 F1 plants generated 1.7-, 1.0-, and 0.7-kbp bands, irrespective of the plant’s male fertility (Fig 1A). The 1.0-kbp and 0.7-kbp are identical to those of seed parental line TA-33BB-CMS. Therefore, the 1.7-kbp band is likely derived from pollen parents ‘Fukkoku-ouba’ #1 and #2. Previously, we named the electrophoretic pattern with a single 1.7-kbp band as pattern 5 [36]. Thus, all 25 F1 plants were heterozygous for pattern 4 and pattern 5. Therefore, no association with *Rf1* was suggested.

To determine the allelic frequency of s17 in ‘Fukkoku-ouba’, we examined 32 ‘Fukkoku-ouba’ plants and found that all were homozygous for the pattern 5 *rf1* marker (Fig 1B). We next examined the copy number of the *orf20*-like gene in ‘Fukkoku-ouba’. A probe was prepared from a DNA fragment of the 3' untranslated region (UTR) of *orf20*, and a DNA gel blot analysis was conducted. Two arbitrarily selected ‘Fukkoku-ouba’ plants from the 32 pattern-5 homozygous plants were subjected to this analysis. As shown in Fig 1C, a single 5.9 kbp signal band appeared, indicating that the pattern-5 *rf1* variant in ‘Fukkoku-ouba’ has a single *orf20*-like gene. Altogether, it seems likely that the ‘Fukkoku-ouba’ *Rf1* locus is fixed with a non-restoring allele of the pattern-5 variant with a single *orf20*-like gene.

**Fig 2:**
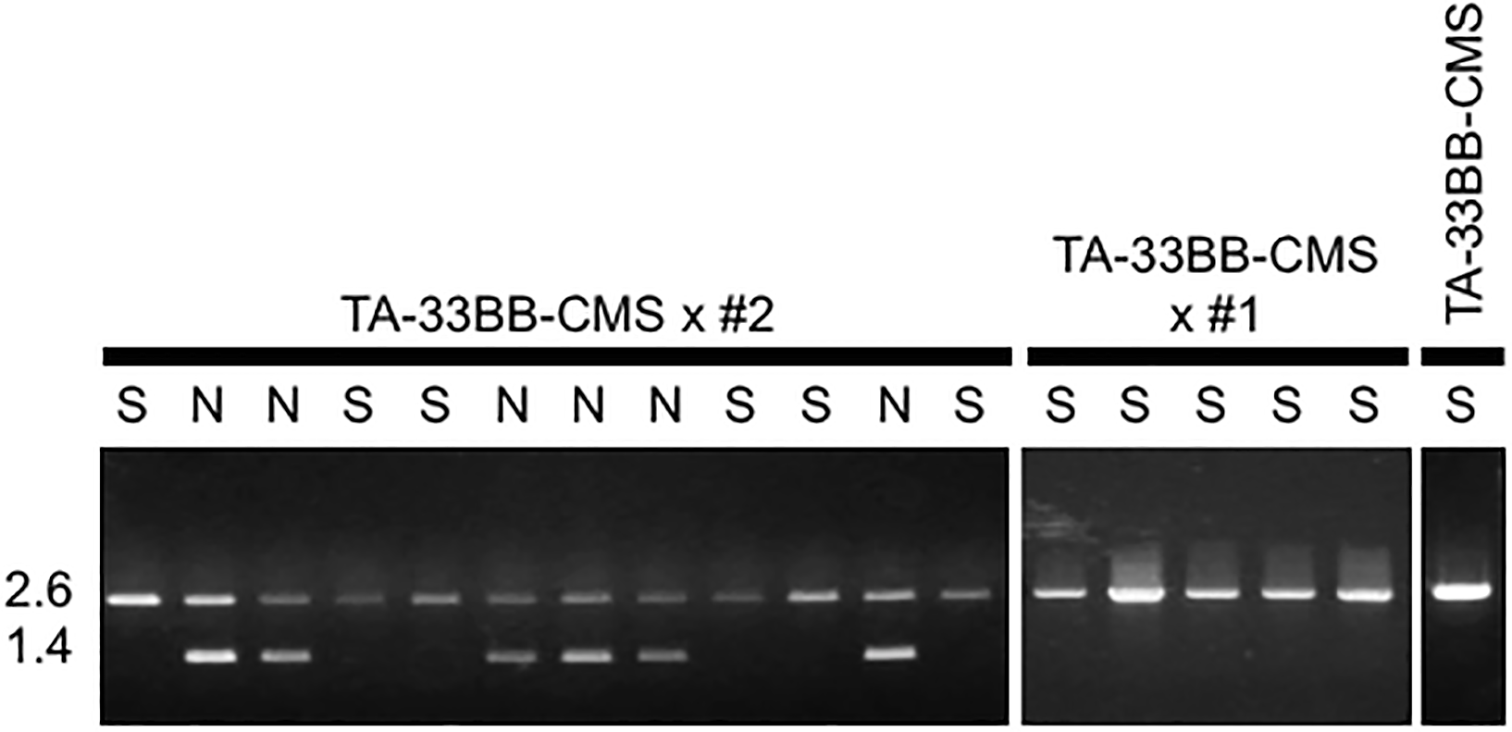
Marker analysis of o7 in F1 plants. Agarose gel electrophoresis of o7 amplified fragments derived from representative F1 plants of the cross TA-33BB-CMS x ‘Fukkoku-ouba’ #2 and TA-33BB-CMS x ‘Fukkoku-ouba’ #1, and a TA-33BB-CMS plant. Male fertility of each plant is indicated as S, completely sterile, or N, normal. Size markers are shown on the left (kbp).

We next tested the association of *Rf2* with fertility restoration using an *Rf2*-linked DNA fragment length polymorphism marker, o7. When the F1 plants derived from the #2 pollen parent were investigated, all seven normal plants amplified 2.6- and 1.4-kbp PCR bands (Fig 2 and Table 3). The 2.6 kbp band was shared with TA-33BB-CMS, but the 1.4 kbp band was unique. We assigned the 2.6 kbp band to the TA-33BB-CMS-type allele and the 1.4 kbp band to the ‘Fukkoku-ouba’-type allele. In contrast, the seven completely sterile plants were homozygous for the TA-33BB-CMS-type allele. No ‘Fukkoku-ouba’-type allele was seen in TA-33BB-CMS x #1 (Fig 2). Thus, we assumed that the #2 pollen parent was heterozygous for o7. Based on male fertility segregation in the F1, an association of *Rf* linked to o7 was suggested (*p* = 1; Fisher’s exact test).

**Table 3:** Segregation of o7 marker types and male fertility in TA-33BB-CMS x ‘Fukkoku-ouba’ #2.

Male fertility^2^	Marker type^1^		Total
	FT	TT	
N	7	0	7
Sf	0	0	0
CS	0	7	7
Total	7	7	14

^1^F, ‘Fukkoku-ouba’-type allele; and T, TA-33BB-CMS-type allele.

^2^N, normal; Sf, semi-fertile; and CS, completely sterile.

To confirm the association of o7-linked *Rf* on fertility restoration and the independence of ‘Fukkoku-ouba’ *rf1*, we selected a fully fertile plant from the F1 of TA-33BB-CMS x #2 and identified this plant as 14–76. We crossed 14–76 with TA-33BB-CMS. From the resultant B1 population (22 plants), the plant with the best pollen fertility was selected for backcrossing with TA-33BB-CMS, thereby yielding a B2 population (16 plants). We examined male fertility and the segregation of o7- and s17 markers in the B1 and B2 populations (Table 4).

**Table 4:** Segregation of male fertility and the o7- and s17-marker-types in the B1 and B2 populations.

Male fertility^1^	B1					B2				
	FT^2^		TT^2^		Total	FT^2^		TT^2^		Total
	45^3^	44^3^	45^3^	44^3^		45^3^	44^3^	45^3^	44^3^	
N	2	0	0	0	2	2	0	0	0	2
Sf	3	3	1	0	7	1	2	0	0	3
CS	0	3	4	6	13	0	1	6	4	11
Total	5	6	5	6	22	3	3	6	4	16

^1^N, normal; Sf, semi-fertile; and CS, completely sterile.

^2^o7 marker type: F, ‘Fukkoku-ouba’-type allele; and T, TA-33BB-CMS-type allele.

^3^s17 marker type: 4, pattern 4; and 5, pattern 5

The observed segregation of o7 did not significantly differ from those expected from a 1:1 ratio in both the B1 and the B2 populations (*p* = 1 and *p* = 0.72 for B1 and B2, respectively; Fisher’s exact test). Similarly, segregation of s17 appeared to follow a 1:1 ratio (*p* = 1 and *p* = 0.832 for B1 and B2, respectively; Fisher’s exact test). Segregation of fertility restoration (fully or partially restored plants vs. completely sterile plants) can be explained by a single gene (*p* = 0.523 and *p* = 0.21 for B1 and B2, respectively; Fisher’s exact test). We tested two null hypotheses that the marker types of s17 and o7 were independent of male fertility. Although the former was not rejected (*p* = 0.192 and *p* = 1 for B1 for B2, respectively; Fisher’s exact test), the latter was (*p* = 0.006 and *p* = 0.001 for B1 and B2, respectively; Fisher’s exact test). Again, association of the o7-linked *Rf* was suggested; however, we thought the appearance of the semi-fertile phenotype in the B1 and the B2 populations was puzzling because no such phenotype was observed in the F1. Thus, we hypothesized that segregation of another gene associated with fertility restoration must occur.

### Modifier gene in ‘Fukkoku-ouba’

After proposing the existence of another genetic factor that affects the degree of fertility restoration, we selfed 14–76 and obtained an F2 population of 74 plants. To minimize the environmental effects, the F2 plants were grown in an air-conditioned greenhouse. We genotyped the F2 plants using the o7 and s17 markers (Tables 5 and 6). The observed segregation did not deviate significantly from the expected ratio (*p* = 0.263 and *p* = 0.533 for o7 and s17, respectively; Fisher’s exact test). Segregation of fertility restoration fit a 3:1 ratio (*p* = 0.140; chi-square test). The independence of o7 marker type on fertility restoration was rejected (*p* = 2.55 x 10^−11^; Fisher’s exact test), supporting the association of an o7-linked *Rf*.

**Table 5:** Segregation of o7 marker types in an F2 population.

Male fertility^1^	o7 marker type^2^			Total
	FF	FT	TT	
N	5	29	1	50
Sf	7	6	2	
CS	1	3	20	24
Total	13	38	23	74

^1^N, normal; Sf, semi-fertile; and CS, completely sterile.

^2^F, ‘Fukkoku-ouba’-type allele; and T, TA-33BB-CMS-type allele.

**Table 6:** Segregation of s17 marker types in an F2 population.

Male fertility^1^	s17 marker type^2^			Total
	55	45	44	
N	10	24	1	50
Sf	1	9	5	
CS	5	9	10	24
Total	16	42	16	74

^1^N, normal; Sf, semi-fertile; and CS, completely sterile.

^2^4, pattern 4; and 5, pattern 5

We tested another null hypothesis that the s17 marker type and fertility restoration were independent, and the result was marginal (*p* = 0.0137; Fisher’s exact test). To investigate the cause of this result, we examined the correlation of marker types and phenotypes in detail (Table 7). We focused our analysis on the plant group with the ‘Fukkoku-ouba’-type o7 allele. In this group, no completely sterile plant was found when the plant had the pattern 5 s17 allele (Table 7) (*p* = 0.00084; Fisher’s exact test). In addition, of the fully or partially restored plants in this group (i.e. normal and semi-fertile), the number of normal plants increased when they had the pattern 5 s17 allele compared with those without this allele (*p* = 0.00424; Fisher’s exact test). Thus, male fertility appeared to increase when the plant had the pattern 5 s17 allele. Our focus next moved to the plant group without the ‘Fukkoku-ouba’-type o7 allele. The null hypothesis that the s17 type was independent of fertility restoration was not rejected (*p* = 0.539; Fisher’s exact test). In short, there may be a genetic factor that does not restore male fertility by itself but strengthens the effect of the o7-linked *Rf*. Genetic linkage between this genetic factor and s17 was obvious.

**Table 7:** Marker types and male fertility in F2 population.

Male fertility^2^	o7 and s17 marker types^1^						Total
	F_			TT			
	55	45	44	55	45	44	
N	9	24	1	1	0	0	35
Sf	1	7	5	0	2	0	15
CS	0	0	4	5	9	6	24
Total	10	31	10	6	11	6	74

^1^F, ‘Fukkoku-ouba’-type allele of o7; T, TA-33BB-CMS-type allele of o7; 4, pattern 4 of s17; and 5, pattern 5 of s17.

^2^N, normal; Sf, semi-fertile; and CS, completely sterile.

### Molecular organization of ‘Fukkoku-ouba’ *rf1*

**Fig 3:**
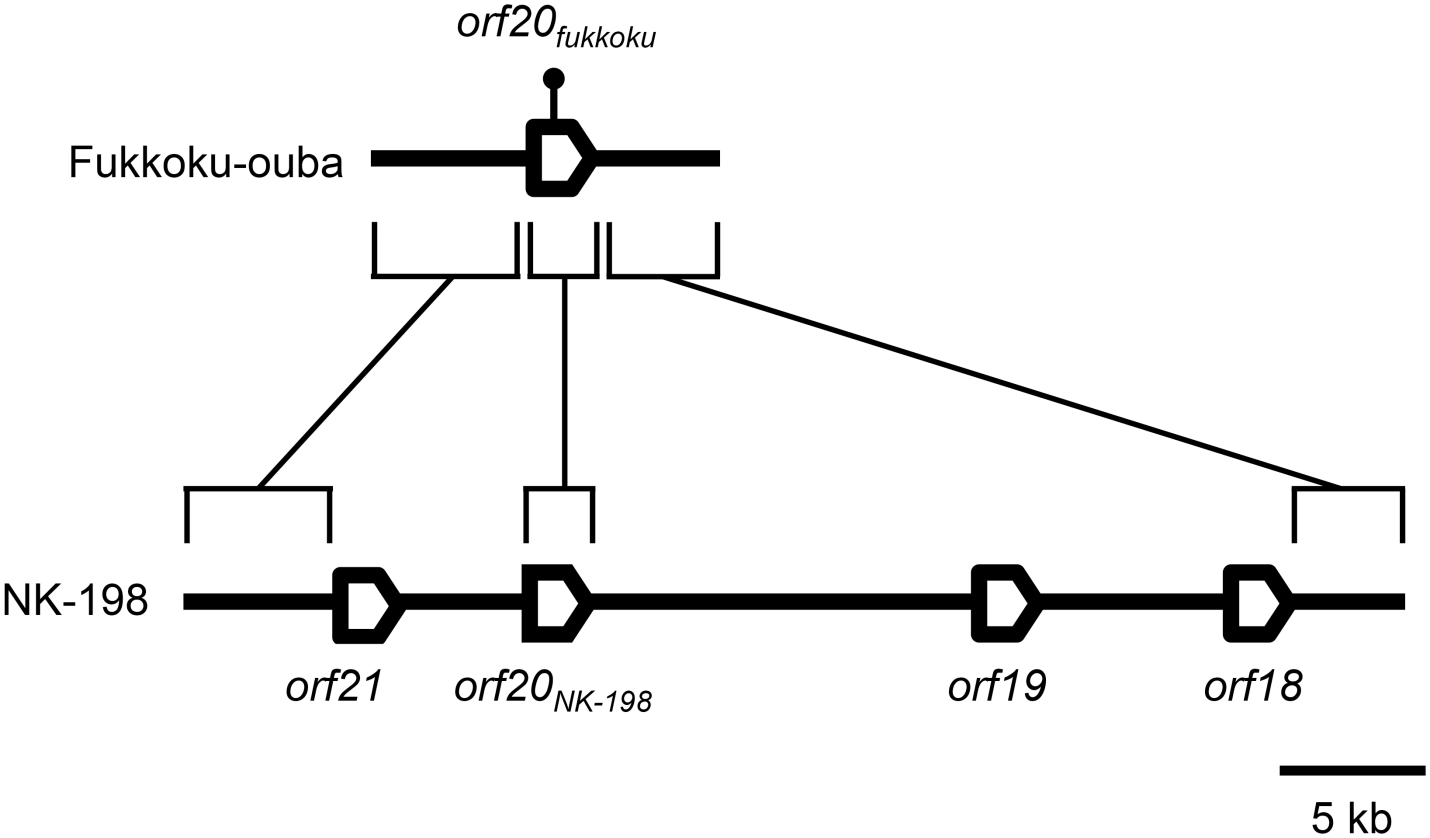
Comparison of the genomic organization between ‘Fukkoku-ouba’ *rf1* and NK-198 *Rf1*. ‘Fukkoku-ouba’ has a single copy of an *orf20*-like gene whereas homologs are clustered in NK-198. Boxes indicate genes (introns are not shown). Brackets denote the most similar regions. A single nucleotide substitution in exon 2 of *orf20*_*fukkoku*_ is shown by a lollipop. The scale bar is shown below. The NK-198 *Rf1* sequences correspond to DDBJ/EMBL/GenBank accession numbers AB646135 and AB646133.

Our data suggested that ‘Fukkoku-ouba’ *rf1* was a non-restoring allele. Therefore, we raised the question of whether ‘Fukkoku-ouba’ *rf1* was identical to either of the previously identified non-restoring *rf1* molecular variants in sugar beet. DNA gel blot analysis (Fig 1C) indicated that ‘Fukkoku-ouba’ has a single copy of an *orf20*-like gene, leading us to conduct a PCR-based strategy to nucleotide sequence the ‘Fukkoku-ouba’ *rf1* region. A nucleotide sequence of 9871 bp was obtained. Comparison with the previously identified *orf20*-like genes enabled us to determine exon-intron boundaries, and an *orf20*-like gene was identified. Hereafter, we refer to the identified gene as *orf20*_*fukkoku*_. To our surprise, the nucleotide sequence of *orf20*_*fukkoku*_ matched best with *orf20*, which had been previously identified as the responsible gene for fertility restoration from the restorer line NK-198 (hereafter *orf20*_*NK-198*_) [30]. Comparison of the *orf20*_*fukkoku*_ and *orf20*_*NK-198*_ sequences revealed that the exons and introns of the two genes are identical, except for a single nucleotide substitution that changes the amino acid specificity (TCT to TTT; Ser299 to Phe299) (S1 Fig and the schematic illustration in Fig 3). Therefore, we further compared the upstream- and downstream regions of *orf20*_*fukkoku*_ with those of the *orf20*-like genes in NK-198. The upstream region was most similar to *orf21*, one of the four *orf20*-like genes in NK-198 (Fig 3). Of the 4187 nucleotide residues, there were one substitution and two indels (S2 Fig). On the other hand, the *orf20*_*fukkoku*_ downstream sequence is most similar to that of *orf18*, another *orf20*-like gene in NK-198 (Fig 3 and S3 Fig). Matsuhira et al. [30] reported that *orf21* and *orf18* were incapable of fertility restoration in their transgenic experiment.

### Protein products of *orf20*_*fukkoku*_

**Fig 4:**
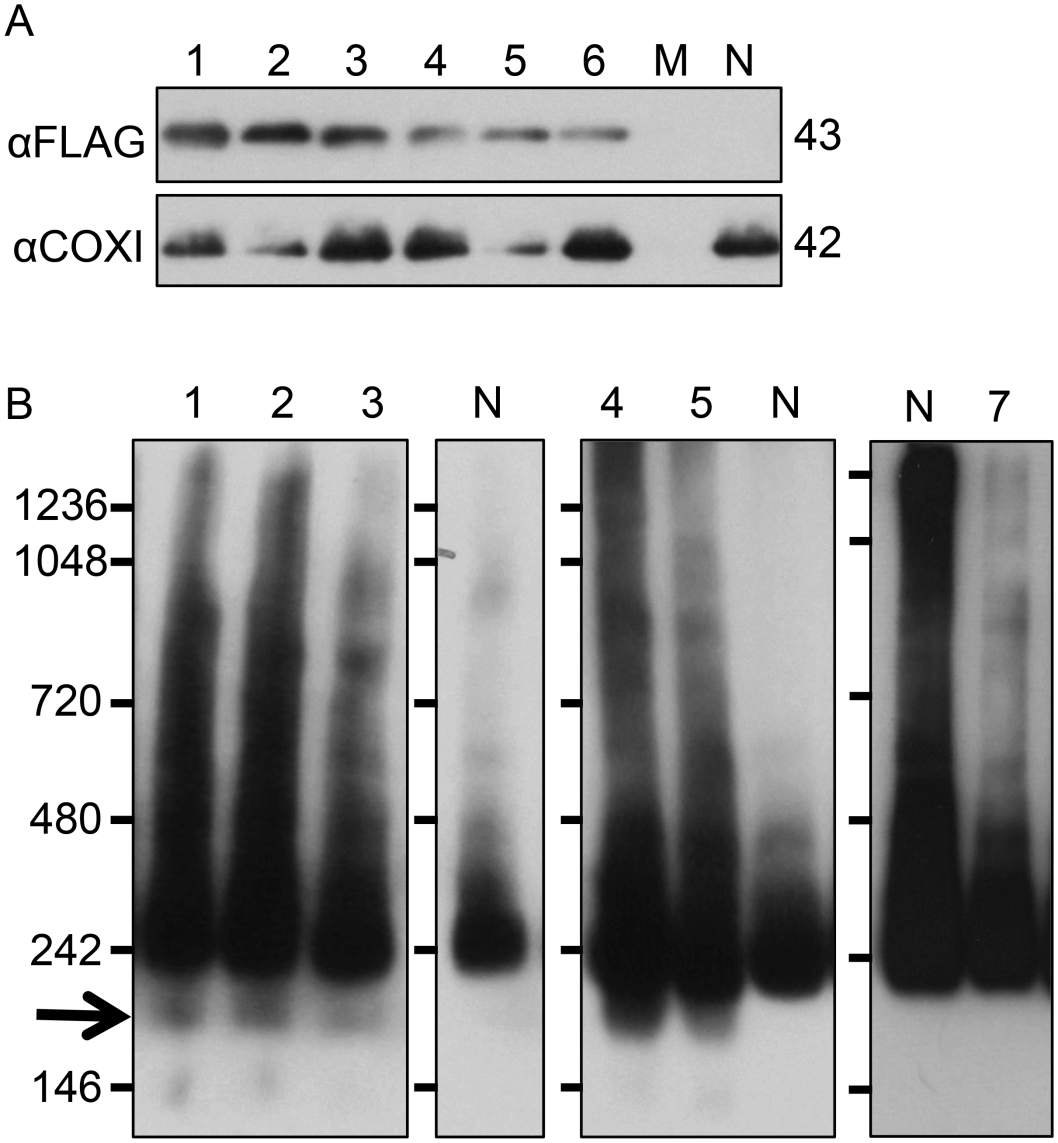
Immunoblot analysis of transgenic suspension cells expressing FLAG-fused *orf20*_*fukkoku*_. (A) Total cellular proteins were separated by SDS-PAGE and probed with αFLAG or αCOXI. Lanes 1 to 6 are transgenic cell lines expressing FLAG-fused *orf20*_*fukkoku*_. Lane M and lane N are the size marker (that does not react with αFLAG or αCOXI) and non-transgenic cells, respectively. Molecular masses are shown on the right. (B) Mitochondria were electrophoresed by BN-PAGE and probed with αpreSATP6. Lanes 1–3, lanes 4–5, and lane 7 are transgenic cell lines expressing FLAG-fused *orf20*_*fukkoku*_, FLAG-fused *orf20*_*NK-198*_, and FLAG-fused *orf20L*, respectively. Lane N is the non-transgenic cells control. Size markers are shown on the left. An arrow indicates the position of the 200-kDa signal band.

To determine whether the Ser299-to-Phe299 substitution in *orf20*_*fukkoku*_ affects the function of protein products, we prepared a construct in which expression of FLAG-fused *orf20*_*fukkoku*_ was driven by the cauliflower mosaic virus (CaMV) 35S promoter. The use of the CaMV promoter was necessary because the expression of *orf20*-like genes is very weak in non-anther organs and tissues, whereas *preSatp6* is expressed constitutively [32]. As a negative control, FLAG-fused *orf20L* (a gene cloned from a sugar beet maintainer line) was used [32]. The transgene was introduced into sugar beet suspension cells that were derived from an Owen CMS line via *Agrobacterium*. We obtained several cell lines expressing the transgene, as judged by immunoblot analysis using αFLAG (Fig 4A). We extracted mitochondria from three of the obtained cell lines for BN-PAGE. Protein complexes containing the preSATP6 protein on the blot were visualized by immunoblot analysis using αpreSATP6 (Fig 4B). Smeared images appeared on the blot, in which the most intense signal was a 250 kDa band. In addition, a signal band at 200 kDa was conspicuous. The 200-kDa signal band was also obtained from transgenic suspension cells expressing the FLAG-fused *orf20*_*NK-198*_ transgene, but not from those expressing FLAG-fused *orf20L* or from non-transgenic cells (Fig 4B). The obtained band patterns were not as clear as those from anther samples (see below). This may be due to differences between suspension cells and anther tissues in terms of physiological condition including mitochondrial number and activity [32]. The 200-kDa signal band has been shown to contain both preSATP6 and ORF20_NK-198_ proteins, so it is the hallmark for protein-protein interaction between preSATP6 and ORF20_NK-198_ [32]. Because *orf20*_*fukkoku*_ produced the same 200-kDa signal band, its protein product likely has the same function as *orf20*_*NK-198*_.

### ‘Fukkoku-ouba’ *rf1* has a very small effect on the 250 kDa complex in anthers

The fertility-restoring potential of *orf20*_*fukkoku*_ seemed to be in contradiction with the results of our genetic analysis. Therefore, we examined the molecular effects of ‘Fukkoku-ouba’ *rf1* on anthers. A male sterile plant was selected from the TA-33BB-CMS x ‘Fukkoku-ouba’ #2 cross (thus, lacking o7-linked *Rf*). Using this male sterile plant as the seed parent, we backcrossed twice with the recurrent pollen parental line TA-33BB-O (maintainer line of TA-33BB-CMS) and selected heterozygous plants for the s17-marker type in every generation. The obtained B2 plants were completely male sterile. The B2 plants heterozygous for s17 were named B2-Fukkoku.

**Fig 5:**
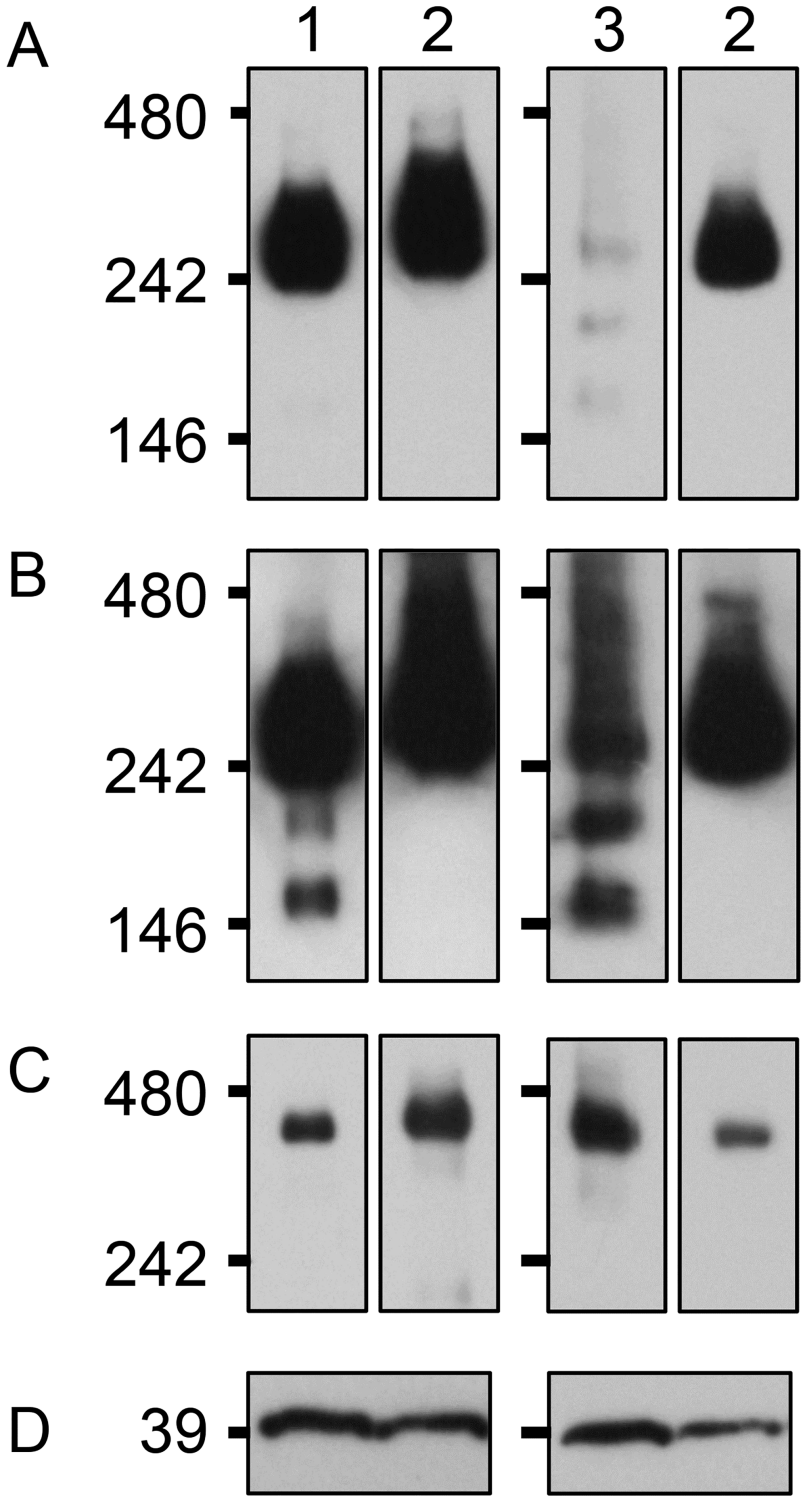
Protein complexes containing preSATP6 in anthers. (A) Anthers of a plant heterozygous for ‘Fukkoku-ouba’ *rf1* (lane 1), TA-33BB-CMS (lane 2), and NK-198 (lane 3) were subjected to BN-PAGE, and the protein complexes were blotted onto membranes and probed with αpreSATP6. Size markers are shown on the left. Exposure time was 1 min. (B) The same blot as panel A but exposed for 2 hr. (C) The same blot as panel A but probed with αCOXI. (D) Anthers of a plant heterozygous for ‘Fukkoku-ouba’ *rf1* (lane 1), TA-33BB-CMS (lane 2), and NK-198 (lane 3) were subjected to SDS-PAGE and αpreSATP6 was used for immunodetection.

The preSATP6-protein complex in the anthers of B2-Fukkoku was investigated by immunoblot analysis. Immature anthers (most of them were in meiosis or the tetrad stage) of B2-Fukkoku were subjected to immunoblot analysis using αpreSATP6 (Fig 5). Combined with BN-PAGE, a 250-kDa signal band appeared on the blot. Compared with TA-33BB-CMS, the signal intensity for B2-Fukkoku was slightly reduced or indistinguishable when the exposure time for signal detection was short (Fig 5A). On the other hand, the intensity of the 250-kDa band was highly reduced in fully fertile NK-198 and additional 200- and 150 kDa bands appeared. However, under longer exposure times, the 200- and 150-kDa bands appeared in samples from the anthers of B2-Fukkoku but not in TA-33BB-CMS (Fig 5B). We examined the total amount of preSATP6 protein by immunoblot combined with SDS-PAGE and found that the accumulation of preSATP6 protein was at a similar level among B2-Fukkoku, TA-33BB-CMS, and NK-198 (Fig 5D).

### Transcript abundance of *orf20*_*fukkoku*_ is highly reduced in anthers

We focused our analysis on the transcript abundance of *orf20*_*fukkoku*_ in anthers of B2-Fukkoku and NK-198 plants using quantitative reverse transcription PCR (qRT-PCR). The nucleotide sequences of *orf20*-like genes are so similar that it was impossible to design specific primer sets for each homolog (see Fig 3). The results are summarized in Table 8. Transcript abundance of *orf20*-like genes was highest during meiosis or the tetrad stage and then decreased during the microspore stage in both NK-198 and the B2-Fukkoku. Transcript abundance of B2-Fukkoku was one-third to one-sixth that of NK-198. These differences are statistically significant at all anther developmental stages (Table 8). It should be noted that Matsuhira et al. [30] suggested that *orf20*_*NK-198*_ transcripts were the most abundant in NK-198 anthers among the four *orf20*-like genes based on cDNA sequence frequency.

**Table 8:** Summary of qRT-PCR (mean ± SD) (n = 3).

Anther developmental stage	Reference gene	NK-198	B2-Fukkoku	*t*-test
Meiosis	*Actin*	0.997±0.248	0.256±0.046	*p* = 0.004
	*ef1α*	0.320±0.068	0.056±0.014	*p* = 0.001
Tetrad	*Actin*	0.897±0.174	0.200±0.023	*p* = 0.001
	*ef1α*	0.327±0.047	0.050±0.007	*p* = 0.0003
Microspore	*Actin*	0.444±0.078	0.103±0.017	*p* = 0.001
	*ef1α*	0.222±0.036	0.028±0.002	*p* = 0.0004

## Discussion

Japanese leaf beet was first introduced from China and, more recently, from Europe [44]; no endemic wild *Beta* species is found in Japan. Therefore, the fertility restoring genotype reported here was probably derived from the introduced genotype. In leaf beet, the Owen cytoplasm is rare. Cheng et al. [44] reported that Owen cytoplasm was found from only one French and two Chinese accessions among 77 leaf beet accessions collected throughout the Eurasian and African continents. This research group also reported no Owen cytoplasm in their Japanese leaf beet collections, including ‘Fukkoku-ouba’ [44]. Very little is known about the frequency and distribution of the fertility-restoring genotype in leaf beet, as far as we know. Since the fertility-restoring genotype is frequent in sugar beet and wild beet despite the low frequency of Owen cytoplasm [13] [45], this might be also the case in leaf beet. The significance of the fertility-restoring genotype in beets remains an open question.

Here, we pose a genetic model as follows: we propose that ‘Fukkoku-ouba’ #2 is heterozygous for the o7-linked weak *Rf* and homozygous for the s17-linked modifier. TA-33BB-CMS (and TA-33BB-O) are proposed to be homozygous recessives at both loci. Accordingly, in their F1, segregation of the the o7-linked *Rf* occurred but the s17-linked modifier was held as a heterozygote. Because the modifier strengthens the o7-linked *Rf*, the phenotypes in the F1 were either normal or completely sterile according to the presence or absence of the o7-linked *Rf*. In subsequent generations, such as B1 and B2, the o7-linked *Rf* and the s17-linked modifier segregated, and plants with only the o7-linked *Rf* expressed a semi-fertile or occasionally completely sterile condition in the absence of an s17-linked modifier. This model appears to be consistent with all our observations.

The o7-linked *Rf* is weak as judged by the phenotype of plants that had only this *Rf*. Such plants were semi-fertile at the best in the B1 and B2 (see FT/44 plants in Table 4). In the F2, only one plant was normal whereas the remaining nine plants were either semi-fertile or completely sterile (see F_/44 plants in Table 7). The weak effect and map position of the o7-linked *Rf* coincides with that of *Rf2* reported by Honma et al. [33]. Thus, we think it likely that the o7-linked *Rf* is an allele of *Rf2*, but this hypothesis is still inconclusive. Hjerdin-Panagopoulos *et al*. [46] proposed that Owen’s *Z* may not be a single gene but two linked *Rf*s. Considering this proposal, the relationship among *Rf*s on chromosome 4 is still unclear.

We incorporated a modifier into our genetic model because the degree of fertility restoration by the o7-linked *Rf* was higher in the presence of the ‘Fukkoku-ouba’ s17 allele. We consider this gene as a modifier because fertility restoration was hardly seen in the absence of the o7-linked *Rf* (compare TT/45 and TT/44 in Table 4, and TT/_5 and TT/44 in Table 7). A normal fertile plant in the F2 appeared to be exceptional, but this might be a recombinant between o7 and the o7-linked *Rf*. Modifier genes for *Rf* have been proposed for several plant species such as maize [10], sunflower [47], pepper [48], and wheat [49], but few details are known. In sugar beet, Hogaboam reported a modifier, *Sh*, that strengthens the effect of *Rf* linked to *M*, which is now known to map to chromosome 4 [16]. The modifier gene identified in this study is very similar to *Sh* because both showed epistasis to a weak *Rf* on chromosome 4. It is possible that the s17-linked modifier is an allele of *Sh*, but further study is necessary to confirm this hypothesis.

Our molecular analysis of ‘Fukkoku-ouba’ *rf1* aimed to clarify whether its gene organization is identical to either of the known *rf1*-molecular variants or is novel. Analysis of the ‘Fukkoku-ouba’ *rf1* nucleotide sequence revealed a very high homology to *orf20*_*NK-198*_, a gene responsible for fertility restoration. Only one nucleotide substitution differed in the coding regions of *orf20*_*fukkoku*_ and *orf20*_*NK-198*_. This finding seems to contradict our genetic data indicating the non-restoring action of ‘Fukkoku-ouba’ *rf1*. Moreover, the results of transgenic suspension-cell analysis showed that *orf20*_*fukkoku*_ could give rise to a characteristic signature for protein-protein interaction with preSATP6 (i.e. a 200-kDa signal band). Appearance of the 200-kDa signal band was never seen in *orf20L*, a gene from a maintainer sugar beet line [32]. These data suggest that the ORF20_fukkoku_ protein may function in the same manner as ORF20_NK-198_. Although we saw no evidence for the nucleotide substitution in the coding region affecting function, the possibility remains.

The results of transgenic suspension cell analysis are consistent with the occurrence of the 200-kDa band in B2-Fukkoku anthers; however, preSATP6 complexes in anthers are clearly quantitatively different between B2-Fukkoku and NK-198. Although both B2-Fukkoku and NK-198 anthers displayed the 200 kDa signal band, the signal intensity for B2-Fukkoku was weaker than for NK-198. On the other hand, a decrease in the signal intensity of the 250-kDa band was conspicuous in NK-198 but not evident for B2-Fukkoku. In accordance with these observations, the results of qRT-PCR analyses indicated a smaller amount of *orf20*_*fukkoku*_ mRNA, suggesting an association with the reduced signal intensity of the 200-kDa signal band. Altogether, our molecular data suggest that *orf20*_*fukkoku*_ is a hypomorphic (leaky) allele but not an amorphic one.

The 250-kDa complex is thought to be a homo-oligomer of preSATP6 [27] [32], and oligomer formation of *S-orf* protein products is well known in several plant species [28] [29]. Upon fertility restoration, the oligomer disappears as a consequence of reduced levels of *S-orf* protein products [50] [51], supporting a notion that such an oligomer is associated with the male sterility phenotype. Sugar beet *Rf1* reduces the oligomer form, but accumulation of preSATP6 protein products is almost unchanged [27] [32], indicating the importance of the homo-oligomer form. In this regard, the male sterility phenotype of B2-Fukkoku is reasonable considering the large amount of 250-kDa complex in its anthers. On the other hand, because the signal pattern of preSATP6 on the BN-PAGE blot was clearly different from TA-33BB-CMS, the effect of the 250-kDa complex in B2-Fukkoku may be partially reduced such that it helps another *Rf* to restore fertility. This notion prompts us to hypothesize that *orf20*_*fukkoku*_ is the best candidate for the s17-linked modifier, but clearly this idea needs to be supported by additional experiments. Molecular characterization of *Rf2* would help to identify the genetic and molecular bases for the epistasis.

The molecular organization of ‘Fukkoku-ouba’ *rf1* suggests its close evolutionary relationship to NK-198 *Rf1* (see Fig 3). At a glance, deletion of the *orf18-orf19* and *orf21* regions in NK-198 *Rf1* appeared to result in the ‘Fukkoku-ouba’ *rf1*, but this notion remains speculative and requires additional samples to trace the evolutionary events. These presumptive deletions appear to be functionally significant and might be associated with the gene expression pattern of *orf20*_*fukkoku*_. Variation in the copy number of gene arrays is typical in plant *Rf*s, e.g. [52] [53]. Notably, all such reported arrays consist of PPR-encoding genes. Considering that the protein products of *orf20*-like genes are not PPR proteins, the similarity in the pattern of diversification is interesting. As pointed out by Matsuhira et al. [30], the evolutionary mechanism of some *Rf*s may be common irrespective of the encoded protein. We are seeking additional examples that support this notion.

What are the implications of modifiers in hybrid breeding? In crops such as sugar beet, the amount of F1 seeds obtained is sometimes insufficient because they are set on inbred lines expressing inbreeding depression. In such cases, breeders use three-way crosses in which a male sterile F1 plant is crossed with another pollinator parent. In this case, the F1 plants should be male sterile. If one of the parental lines of the F1 has a weak *Rf* and the other has its modifier, then male fertility is restored in the resultant F1, a situation that reduces the quality of the hybrid. At present, elimination of *Rf* largely depends on test crosses that may dismiss a weak *Rf* if the selecting condition is unfavorable for male fertility restoration. In addition, test crosses cannot eliminate modifiers in principle. We have been less aware of modifiers in breeding programs. Of the three molecular variants of *rf1* in sugar beet, two remain to be characterized at the molecular level regarding the properties of their translation products and transcript abundance. These data could help in understanding the potential of these less known molecular variants of *rf1* to become modifiers.

## Conclusions

A Japanese leaf beet accession ‘Fukkoku-ouba’ has a fertility restoring genotype for Owen-type CMS, which is used for hybrid seed production in sugar beet. Two DNA markers linked to two sugar beet *Rf*s were used for genetic analysis. Fertility restoration was associated with o7, one of the DNA markers that is linked to *Rf2* on chromosome 4. Another *Rf* locus (i.e. *Rf1* on chromosome 3) in ‘Fukkoku-ouba’ appeared to be occupied by a non-restoring allele. In addition to the o7-linked *Rf*, another genetic factor that strengthens the action of this *Rf* but apparently is unable to restore fertility by itself was proposed. The assumed gene was linked to s17, the other DNA marker linked to *Rf1*. As the ‘Fukkoku-ouba’ *rf1* was investigated in detail at the molecular level, it appeared to be a hypomorphic allele but not an amorphic one for the following reasons: (1) the gene-coding region of ‘Fukkoku-ouba’ *rf1* is highly similar to that of a restoring *Rf1* but the up- and downstream regions differ; (2) the protein products of ‘Fukkoku-ouba’ *rf1* ectopically expressed in transgenic suspension cells were indistinguishable with restoring *Rf1* in terms of their molecular interaction with a CMS-associated mitochondrial protein; (3) in anthers, the amount of ‘Fukkoku-ouba’ *rf1* mRNA was highly reduced compared to restoring *Rf1*; (4) molecular interaction with a CMS-associated mitochondrial protein was observed in anthers expressing ‘Fukkoku-ouba’ *rf1* but to a lesser degree than for restoring *Rf1*, leaving a homo-oligomer form of CMS-associated mitochondrial protein remaining at a level comparable to that of the CMS line. Provided that the homo-oligomer form is associated with a CMS phenotype as seen in some other plant CMSs, the inability of ‘Fukkoku-ouba’ *rf1* to restore male fertility is a reasonable assumption. On the other hand, detection of the molecular interaction between ‘Fukkoku-ouba’ *rf1* and a CMS-associated mitochondrial protein at a lower quantity may suggest that ‘Fukkoku-ouba’ *rf1* slightly reduces the action of the homo-oligomer form of CMS-associated mitochondrial protein to a level insufficient to restore male fertility but boosts the action of another *Rf*. This hypothesis implies that ‘Fukkoku-ouba’ *rf1* is a strong candidate for the modifier detected in our genetic analysis.

## Supporting information

S1 Fig Alignment of nucleotide sequences between *orf20*_*fukkoku*_ and *orf20*_*NK-198*_. (PDF)

S2 Fig Alignment of nucleotide sequences between *orf20*_*fukkoku*_ and *orf21*. (PDF)

S3 Fig Alignment of nucleotide sequences between *orf20*_*fukkoku*_ and *orf18*. (PDF)

S1 File (TIF)

S2 File (TIF)

S1 Text DNA markers and male-fertility indices of F2 plants derived from the cross TA-33BB-CMS x ‘Fukkoku-ouba’ 14–76. (PDF)
