# Supplementary material for: A fertility-restoring genotype of beet (Beta vulgaris L.) is composed of a weak restorer-of-fertility gene and a modifier gene tightly linked to the Rf1 locus
Source: PLoS One. 2018 Jun 1;13(6):e0198409. doi: 10.1371/journal.pone.0198409 (PMC5983528; doi:10.1371/journal.pone.0198409)
Supplement: S1 Fig — (PDF) [file pone.0198409.s001.pdf]

```
                / Exon 1
fukkoku         ATGGCGTGGTACAGAAATTCAAGGTTTGTCTACAATGCTTTAAAACTCAACTTGCGTTCC 60
ORF20           ATGGCGTGGTACAGAAATTCAAGGTTTGTCTACAATGCTTTAAAACTCAACTTGCGTTCC 60
                ************************************************************

fukkoku         AAAACATTTGGTACTATTCCAACTCCAAGAGTTCATTCGAATTCCTCATCTTTGTTTTAC 120
ORF20           AAAACATTTGGTACTATTCCAACTCCAAGAGTTCATTCGAATTCCTCATCTTTGTTTTAC 120
                ************************************************************

fukkoku         AATCAATCTACTAATAAGTGTAGTGGGTTATTTGGGTCTGCAAAATCTGGGTATTTTAAT 180
ORF20           AATCAATCTACTAATAAGTGTAGTGGGTTATTTGGGTCTGCAAAATCTGGGTATTTTAAT 180
                ************************************************************

fukkoku         GGGTTTAAACATCATCAAGAGATTAGCTCTTTCTCTGGTTTTGCAAGGAGAAATTATCAT 240
ORF20           GGGTTTAAACATCATCAAGAGATTAGCTCTTTCTCTGGTTTTGCAAGGAGAAATTATCAT 240
                ************************************************************

fukkoku         GGTGATAAAACCGAAGTAAGTGTTGAATCATGGCTGGAAAAATTCCTTGTTCCAATTGGA 300
ORF20           GGTGATAAAACCGAAGTAAGTGTTGAATCATGGCTGGAAAAATTCCTTGTTCCAATTGGA 300
                ************************************************************

fukkoku         CTAATCTTGACTTTTGGTATACTTGGTTACCCTCATGTGCACCCAGTAGTTGTGCCATAT 360
ORF20           CTAATCTTGACTTTTGGTATACTTGGTTACCCTCATGTGCACCCAGTAGTTGTGCCATAT 360
                ************************************************************

fukkoku         ACAGGAAGGAAGCATTATGTGCTTATGTCAACAACTCGTGAGAATGAAATTGGAGAAGTT 420
ORF20           ACAGGAAGGAAGCATTATGTGCTTATGTCAACAACTCGTGAGAATGAAATTGGAGAAGTT 420
                ************************************************************

fukkoku         GAGAAGCGGAAAATACAACCTGCTACACACCCTGATACTGATAGGGTTAGGTCAATATTC 480
ORF20           GAGAAGCGGAAAATACAACCTGCTACACACCCTGATACTGATAGGGTTAGGTCAATATTC 480
                ************************************************************

fukkoku         CAACACATTCTTGAATCACTGGAAAGAGAGATTAATCACCATGAACTCGAACTCGAAAGA 540
ORF20           CAACACATTCTTGAATCACTGGAAAGAGAGATTAATCACCATGAACTCGAACTCGAAAGA 540
                ************************************************************

fukkoku         GATGAAACTTTCAAGGAGAAAACCATTTGGAAGGAGGAGACAGTTGATGATAAAGATAGT 600
ORF20           GATGAAACTTTCAAGGAGAAAACCATTTGGAAGGAGGAGACAGTTGATGATAAAGATAGT 600
                ************************************************************

fukkoku         AGGAAGAAGCATAGTGGGGCTAAGATAACTACTAACCATTTGGAAGGGATGAATTGGGAA 660
ORF20           AGGAAGAAGCATAGTGGGGCTAAGATAACTACTAACCATTTGGAAGGGATGAATTGGGAA 660
                ************************************************************

fukkoku         ATTTTCGTTGTTGATAAACCGTTGGTTGAGTCCAGTTATTTATTAGGTGGGAAGATTGTT 720
ORF20           ATTTTCGTTGTTGATAAACCGTTGGTTGAGTCCAGTTATTTATTAGGTGGGAAGATTGTT 720
                ************************************************************

fukkoku         GTTTACACCGGATTGCTCAACCATTGCAACTCTGATGCTGAATTGGCTACAATTATCGCG 780
ORF20           GTTTACACCGGATTGCTCAACCATTGCAACTCTGATGCTGAATTGGCTACAATTATCGCG 780
                ************************************************************
                      / Intron 1
fukkoku         CATCAGGTATATAAAACTATTCATGGGACTCCAATTATGTGCTTAAGCTGATGGTTAATA 840
ORF20           CATCAGGTATATAAAACTATTCATGGGACTCCAATTATGTGCTTAAGCTGATGGTTAATA 840
                ************************************************************
```

```
fukkoku     GAACTATACAAAAAAACTGATGAATTTTAGGTTATCAGATTACATTATGAATGTCATATG 900
ORF20       GAACTATACAAAAAAACTGATGAATTTTAGGTTATCAGATTACATTATGAATGTCATATG 900
            ************************************************************
                                      / Exon 2
fukkoku     TCAATTTGGTGGTATGTATTTGTTAGGTTGGGCATGCTGTGGCTCGACATGAGGCAGAGG 960
ORF20       TCAATTTGGTGGTATGTATTTGTTAGGTTGGGCATGCTGTGGCTCGACATGAGGCAGAGG 960
            ************************************************************

fukkoku     ATTCGACAGCATTTTTCTGGTTGTTAATATCCCTCAACGTGATATTATTTAAAATTCTAT 1020
ORF20       ATTCGACAGCATTTTTCTGGTTGTTAATATCCCTCAACGTGATATTATTTAAAATTCTAT 1020
            ************************************************************

fukkoku     TTACTGAGCCTGAATTTGCCAATGCAAGATCAAAACTACTCTTAAGGCATCCTCTCTTGC 1080
ORF20       TTACTGAGCCTGAATCTGCCAATGCAAGATCAAAACTACTCTTAAGGCATCCTCTCTTGC 1080
            *************** ********************************************
                / Intron 2
fukkoku     AAAAGTAAGTCTCTTACTCTTAAAATGTTTTCTTGATGATTAACAAACATGTGGTACTGC 1140
ORF20       AAAAGTAAGTCTCTTACTCTTAAAATGTTTTCTTGATGATTAACAAACATGTGGTACTGC 1140
            ************************************************************

fukkoku     TACTGCATAACTGTGTTACTGCATCACATATGTTACTGCATAATTGCAAACTATATTACA 1200
ORF20       TACTGCATAACTGTGTTACTGCATCACATATGTTACTGCATAATTGCAAACTATATTACA 1200
            ************************************************************

fukkoku     TGCCCGGACCTAGTAACTTGTTTCATTTGTCAGCGATTTCATTTAGATATCCATTTGAGA 1260
ORF20       TGCCCGGACCTAGTAACTTGTTTCATTTGTCAGCGATTTCATTTAGATATCCATTTGAGA 1260
            ************************************************************

fukkoku     GCAAGTTAAATTTGTATCAAGTTGTGGAATGGAAAAGTAATAGAACTAAATAGAGAGGTG 1320
ORF20       GCAAGTTAAATTTGTATCAAGTTGTGGAATGGAAAAGTAATAGAACTAAATAGAGAGGTG 1320
            ************************************************************

fukkoku     TGATGCTAATAAAATCTAATCCATTACTGAGTAATGGTTTTGGATCGATATATGGATTGC 1380
ORF20       TGATGCTAATAAAATCTAATCCATTACTGAGTAATGGTTTTGGATCGATATATGGATTGC 1380
            ************************************************************

fukkoku     TATATTCCACAGATTCTATCCTTTGTCGCAGATAACATTAAATTTATGTTGTTTATGCAC 1440
ORF20       TATATTCCACAGATTCTATCCTTTGTCGCAGATAACATTAAATTTATGTTGTTTATGCAC 1440
            ************************************************************

fukkoku     ATTTGACACAATAAATTTGAGTTGTGGACTATAATATATATGTGAGTTAGGTAACATATG 1500
ORF20       ATTTGACACAATAAATTTGAGTTGTGGACTATAATATATATGTGAGTTAGGTAACATATG 1500
            ************************************************************
                          / Exon 3
fukkoku     GTGTCAATTTACAGAGTTTGGAAGATTATTCAGGCTAGAGCTCCACAATTACTGCCACGA 1560
ORF20       GTGTCAATTTACAGAGTTTGGAAGATTATTCAGGCTAGAGCTCCACAATTACTGCCACGA 1560
            ************************************************************

fukkoku     ACTATCTGCTTGTCCCTTGTTGGATTGTTTTCCTCGGTGTTTATTCTTTATTATGGTCGG 1620
ORF20       ACTATCTGCTTGTCCCTTGTTGGATTGTTTTCCTCGGTGTTTATTCTTTATTATGGTCGG 1620
            ************************************************************

fukkoku     AAGGAAATAGAAGCAGATCACATTGGAGTGCTTCTGATGGCTTCTGCTGGATACGACCCG 1680
ORF20       AAGGAAATAGAAGCAGATCACATTGGAGTGCTTCTGATGGCTTCTGCTGGATACGACCCG 1680
            ************************************************************

fukkoku     CGAGTTGCACCTCAAGTATATGACAAGCTTGCAAAGCCACTGGGCGACTGGAACTGTTTA 1740
ORF20       CGAGTTGCACCTCAAGTATATGACAAGCTTGCAAAGCCACTGGGCGACTGGAACTGTTTA 1740
            ************************************************************
```

```
fukkoku         GCAACTCATCCATTTGCAAGAATGAGAGCAAAGTTGTTAGCTCGAGCTGATGTTATGAAG 1800
ORF20           GCAACTCATCCATTTGCAAGAATGAGAGCAAAGTTGTTAGCTCGAGCTGATGTTATGAAG 1800
                ************************************************************

fukkoku         GAAGCAGATAAGATATACAATGAAGTTGTAGCAGGACGTGCAATTCAAGGTCTTCAGTAA 1860
ORF20           GAAGCAGATAAGATATACAATGAAGTTGTAGCAGGACGTGCAATTCAAGGTCTTCAGTAA 1860
                ************************************************************
```

S1 Fig. Alignment of nucleotide sequences between *orf20*$_{fukkoku}$ (fukkoku) and *orf20*$_{NK\text{-}198}$ (ORF20). Exonic- and intronic regions are shown. Exon/intron boundaries are indicated by slashes. Nucleotides are numbered from the initiation codon. Asterisks denote matched residues. Single nucleotide substitution (in exon2) is underlined.
