# Supplementary material for: A fertility-restoring genotype of beet (Beta vulgaris L.) is composed of a weak restorer-of-fertility gene and a modifier gene tightly linked to the Rf1 locus
Source: PLoS One. 2018 Jun 1;13(6):e0198409. doi: 10.1371/journal.pone.0198409 (PMC5983528; doi:10.1371/journal.pone.0198409)
Supplement: S2 Fig — (PDF) [file pone.0198409.s002.pdf]

```
fukkoku-5'      TGCATTTGATGATTTGAGTGTTGGTTATTGTCTTCAAGCTTAAATGTGCCTATATTAATA -4129
bvorf21-5'      TGCATTTGATGATTTGAGTGTTGGTTATTGTCTTCAAGCTTAAATGTGCCTATATTAATA -4128
                ************************************************************

fukkoku-5'      CAATTTTTTTAAGGTATATTTGTACATGATTTAATTTCTTAACTAGTATTTGGGTTTGGG -4069
bvorf21-5'      CAATTTTTTTAAGGTATATTTGTACATGATTTAATTTCTTAACTAGTATTTGGGTTTGGG -4068
                ************************************************************

fukkoku-5'      TGTTGCCCCGGGATAGTATTGTTTTCTGAATTTGATATGCATTTTTTTTTTTCATTTCCA -4009
bvorf21-5'      TGTTGCCCCGGGATAGTATTGTTTTCTGAATTTGATATGCATTTTTTTTTT-CATTTCCA -4009
                *************************************************** ********

fukkoku-5'      TTAAAATATACTCCCTCCGTCTCAAAATATAGTTTCCATTTCCATTTTGGGTGTCCCAAA -3949
bvorf21-5'      TTAAAATATACTCCCTCCGTCTCAAAATATAGTTTCCATTTCCATTTTGGGTGTCCCAAA -3949
                ************************************************************

fukkoku-5'      ATATAGTTCCCATATCCCATTTCCATATTTAGTTCCACATTTTTTCGTAATTTGTCTAGA -3889
bvorf21-5'      ATATAGTTCCCATATCCCATTTCCATATTTAGTTCCACATTTTTTCGTAATTTGTCTAGA -3889
                ************************************************************

fukkoku-5'      AAAACCGTGTCCCCTCATTTATTTGCTTCTTGAATTTTGGTTTTTCCTTTGTTTATTCAA -3829
bvorf21-5'      AAAACCGTGTCCCCTCATTTATTTGCTTCTTGAATTTTGGTTTTTCCTTTGTTTATTCAA -3829
                ************************************************************

fukkoku-5'      CCAAAAATGTTACAATTAATGCTCTTCTACCAATTATTCTCCACTCTTTCTCCTAAAATCA -3769
bvorf21-5'      CCAAAAATGTTACAATTAATGCTCTTCTACCAATTATTCTCCACTCTTTCTCCTAAAATCA -3769
                ************************************************************

fukkoku-5'      TCTTTTCCCATACAACATTTATTTAAATAAACAAAAAATTATTATTATTCATCTTATATT -3709
bvorf21-5'      TCTTTTCCCATACAACATTTATTTAAATAAACAAAAAATTATTATTATTCATCTTATATT -3709
                ************************************************************

fukkoku-5'      CTACTTACATAAATTACCGTGAAAAAAGGGAAATGGGAACTATATTTTGGGACGGAGGGA -3649
bvorf21-5'      CTACTTACATAAATTACCGTGAAAAAAGGGAAATGGGAACTATATTTTGGGACGGAGGGA -3649
                ************************************************************

fukkoku-5'      GTATTAAGTAAAACTCAACATTTAAACCATACAAATATAATAATATGGAGACTTAAAGCA -3589
bvorf21-5'      GTATTAAGTAAAACTCAACATTTAAACCATACAAATATAATAATATGGAGACTTAAAGCA -3589
                ************************************************************

fukkoku-5'      TGATTAAAAGTTGGTTGAGATGGTAATTGTGTCATGTATAATAACAAAAGACTACAGGTT -3529
bvorf21-5'      TGATTAAAAGTTGGTTGAGATGGTAATTGTGTCATGTATAATAACAAAAGACTACAGGTT -3529
                ************************************************************

fukkoku-5'      CAAATCTTGTTGCAAGCTTATTTTACTTTTGTTAATTGACATGAGATATATACACATTGG -3469
bvorf21-5'      CAAATCTTGTTGCAAGCTTATTTTACTTTTGTTAATTGACATGAGATATATACACATTGG -3469
                ************************************************************

fukkoku-5'      ACAAATCTACTGAAGTAACAGAGGTGCCACGTGGCGGGTATACATTGTCACGCACACCTT -3409
bvorf21-5'      ACAAATCTACTGAAGTAACAGAGGTGCCACGTGGCGGGTATACATTGTCACGCACACCTT -3409
                ************************************************************

fukkoku-5'      TAAATATATTTGTATAGATGAACTAGAACTGTTTTCTTCTAATAATAAAGAACGATAATG -3349
bvorf21-5'      TAAATATATTTGTATAGATGAACTAGAACTGTTTTCTTCTAATAATAAAGAACGATAATG -3349
                ************************************************************

fukkoku-5'      ATGAAGTTTATTAACAAATGGTATGTAGAAAATGTGATTTGCTTCAACTTTGTAAGGTAA -3289
bvorf21-5'      ATGAAGTTTATTAACAAATGGTATGTAGAAAATGTGATTTGCTTCAACTTTGTAAGGTAA -3289
                ************************************************************
```

```
fukkoku-5'      AATCATGGCAATTAAGCTTTTAGGCGAGAAAATTAAGATCATGAGTATCATTTAATTTGT -3229
bvorf21-5'      AATCATGGCAATTAAGCTTTTAGGCGAGAAAATTAAGATCATGAGTATCATTTAATTTGT -3229
                ************************************************************

fukkoku-5'      AAACTCTTTGTATATCTATCATTTTGTTGCTTATAGTATGCACCATTTTCCTATGTCTTC -3169
bvorf21-5'      AAACTCTTTGTATATCTATCATTTTGTTGCTTATAGTATGCACCATTTTCCTATGTCTTC -3169
                ************************************************************

fukkoku-5'      AAAGCCTCAAAGGAATACTCCATTTTTTTTCTTTTTTTTTTTTGGTCAAGTGGTGATTGG -3109
bvorf21-5'      AAAGCCTCAAAGGAATACTCCATTTTTTTTCTTTTTTTTTTTTGGTCAAGTGGTGATTGG -3109
                ************************************************************

fukkoku-5'      TGAAGTCCCTAGAACCGTGCATTTTGAGTAAAAAAATTAACTGAACAGGTTGAAACTTTT -3049
bvorf21-5'      TGAAGTCCCTAGAACCGTGCATTTTGAGTAAAAAAATTAACTGAACAGGTTGAAACTTTT -3049
                ************************************************************

fukkoku-5'      TACTTGTAAATCATATAAAATTTTTGTTGCATCAGTCAAAAGTGACAGAGGGTGCAAATT -2989
bvorf21-5'      TACTTGTAAATCATATAAAATTTTTGTTGCATCAGTCAAAAGTGACAGAGGGTGCAAATT -2989
                ************************************************************

fukkoku-5'      GCGGAATTACTTCCTCATATACTTTGATGTACCGTTGATATGGTACACTCAATACTAATT -2929
bvorf21-5'      GCGGAATTACTTCCTCATATACTTTGATGTACCGTTGATATGGTACACTCAATACTAATT -2929
                ************************************************************

fukkoku-5'      TATGTTCATTTGCTTATTTGAAATTTTCTTATTTTGTTCATTTGAATGATTTCAAATAAA -2869
bvorf21-5'      TATGTTCATTTGCTTATTTGAAATTTTCTTATTTTGTTCATTTGAATGATTTCAAATAAA -2869
                ************************************************************

fukkoku-5'      TAATAGGCAAAAAAATAGCCTTTAAATGTATCATGCTCGCAACATTTAGGTATATGATAA -2809
bvorf21-5'      TAATAGGCAAAAAAATAGCCTTTAAATGTATCATGCTCGCAACATTTAGGTATATGATAA -2809
                ************************************************************

fukkoku-5'      AATTTATACAAATTTTAATGAAAAAAAAATGTTGAAACAAAAATTTAAGCTAAGCTAAAG -2749
bvorf21-5'      AATTTATACAAATTTTAATGAAAAAAAAATGTTGAAACAAAAATTTAAGCTAAGCTAAAG -2749
                ************************************************************

fukkoku-5'      TTGACTTTTAAGCTCTCTCCTTTTCTGATGCAACAAAGATTTTGTTTTAGCACTAGCTAC -2689
bvorf21-5'      TTGACTTTTAAGCTCTCTCCTTTTCTGATGCAACAAAGATTTTGTTTTAGCACTAGCTAC -2689
                ************************************************************

fukkoku-5'      TTCTTCTATCCCCATAAAATTCGCCATTTGTTTTCTCAAACTCAAATTTCATCAATTTTG -2629
bvorf21-5'      TTCTTCTATCCCCATAAAATTCGCCATTTGTTTTCTCAAACTCAAATTTCATCAATTTTG -2629
                ************************************************************

fukkoku-5'      ATTATATTTTTTCACCATGTAAGAAAATATCTTATCATGTGTTTTTCGTAATCGAGAAAA -2569
bvorf21-5'      ATTATATTTTTTCACCATGTAAGAAAATATCTTATCATGTGTTTTTCGTAATCGAGAAAA -2569
                ************************************************************

fukkoku-5'      CCCATATAGGAAACTGCTTATAAAGCTAGTGAATCAACGAAATATCAACAGGAAAATCAC -2509
bvorf21-5'      CCCATATAGGAAACTGCTTATAAAGCTAGTGAATCAACGAAATATCAACAGGAAAATCAC -2509
                ************************************************************

fukkoku-5'      ATTGTTCATAGGAAAACTCCTATAACATTTGCATACGGTCATTGTTGCACTTTATTCCCT -2449
bvorf21-5'      ATTGTTCATAGGAAAACTCCTATAACATTTGCATACGGTCATTGTTGCACTTTATTCCCT -2449
                ************************************************************

fukkoku-5'      CGGCCCTCGTATATTGATATTAAGTGTATTTTAATCTACGTTTTTTTCTATTGCAACAAT -2389
bvorf21-5'      CGGCCCTCGTATATTGATATTAAGTGTATTTTAATCTACGTTTTTTTCTATTGCAACAAT -2389
                ************************************************************
```

```
fukkoku-5'      TACTACTTTTGATAATTTTACATCTATTGCAACAATTCCATTTTTGGTATAAAAAGCAAC -2329
bvorf21-5'      TACTACTTTTGATAATTTTACATCTATTGCAACAATTCCATTTTTGGTATAAAAAGCAAC -2329
                ************************************************************

fukkoku-5'      ATTTCAGAACAAAGCATGGATTATGCACTAGGGTACCATTCATATAGAAGAATATGATTT -2269
bvorf21-5'      ATTTCAGAACAAAGCATGGATTATGCACTAGGGTACCATTCATATAGAAGAATATGATTT -2269
                ************************************************************

fukkoku-5'      TTTTCAACAACTTTTCAAGATAAAAAAAAGCACACAATATAAAATTAAAGAACATGTAAG -2209
bvorf21-5'      TTTTCAACAACTTTTCAAGATAAAAAAAAGCACACAATATAAAATTAAAGAACATGTAAG -2209
                ************************************************************

fukkoku-5'      GGTGCGTTTTATTCAACTTATTGGCCCTGAACTTATTGGACCTTATCTGAACTGAATTTA -2149
bvorf21-5'      GGTGCGTTTTATTCAACTTATTGGCCCTGAACTTATTGGACCTTATCTGAACTGAATTTA -2149
                ************************************************************

fukkoku-5'      TTGAACCTGAACTGAACTTATTGGAACTTATTAAACCTGATTGGACCTGATTCAACTTAT -2089
bvorf21-5'      TTGAACCTGAACTGAACTTATTGGAACTTATTAAACCTGATTGGACCTGATTCAACTTAT -2089
                ************************************************************

fukkoku-5'      TGGACCTGATTAAACCTGATTGGAACTTATTGGACCTTATTGGAACTTACTGACCTTATT -2029
bvorf21-5'      TGGACCTGATTAAACCTGATTGGAACTTATTGGACCTTATTGGAACTTACTGACCTTATT -2029
                ************************************************************

fukkoku-5'      GAAACCTATTAGACCTTATTGGCCCTGATTGAAACTTATTAGACCTTATTGGACCTGATT -1969
bvorf21-5'      GAAACCTATTAGACCTTATTGGCCCTGATTGAAACTTATTAGACCTTATTGGACCTGATT -1969
                ************************************************************

fukkoku-5'      GAAACTTATTACACCTTATTGGACCTTATTCGACAAAAACATTGACCATGAATAACATAA -1909
bvorf21-5'      GAAACTTATTACACCTTATTGGACCTTATTCGACAAAAACATTGACCATGAATAACATAA -1909
                ************************************************************

fukkoku-5'      ATATTACCACTAACGTAAATACTACCCCTCAAAATTTTTTATGGAGTAATAATTATTATA -1849
bvorf21-5'      ATATTACCACTAACGTAAATACTACCCCTCAAAATTTTTTATGGAGTAATAATTATTATA -1849
                ************************************************************

fukkoku-5'      ATTCGTCCTTTAAAAATAATGATTATTAATTATCTCTTATGATAATTAATTTAATAAAAA -1789
bvorf21-5'      ATTCGTCCTTTAAAAATAATGATTATTAATTATCTCTTATGATAATTAATTTAATAAAAA -1789
                ************************************************************

fukkoku-5'      AATTTACTATTTATATATTTGCCTATACATAACTTTCACCACTAATATGTTTTGATTTTA -1729
bvorf21-5'      AATTTACTATTTATATATTTGCCTATACATAACTTTCACCACTAATATGTTTTGATTTTA -1729
                ************************************************************

fukkoku-5'      TAAAACACTAGTAGAAAATCAAAAGTTAATTAACATTTATTGCTAACAAGTTAAAATTGA -1669
bvorf21-5'      TAAAACACTAGTAGAAAATCAAAAGTTAATTAACATTTATTGCTAACAAGTTAAAATTGA -1669
                ************************************************************

fukkoku-5'      CACATATAAAAAATTAACATTTATTGAAGAGGGTGATGTAGAAGATGAAGAAAGATACCC -1609
bvorf21-5'      CACATATAAAAAATTAACATTTATTGAAGAGGGTGATGTAGAAGATGAAGAAAGATACCC -1609
                ************************************************************

fukkoku-5'      CGATGAAGAAAGATACTCTAGTGATGATAATGAAGCAATCAATTGACAACAATTATGTCT -1549
bvorf21-5'      CGATGAAGAAAGATACTCTAGTGATGATAATGAAGCAATCAATTGACAACAATTATGTCT -1549
                ************************************************************

fukkoku-5'      TTCATTGTTATTAGTAACGAAAACATGTTATCTCTAGTTATTTAAAGACGAATTGCAAAT -1489
bvorf21-5'      TTCATTGTTATTAGTAACGAAAACATGTTATCTCTAGTTATTTAAAGACGAATTGCAAAT -1489
                ************************************************************
```

```
fukkoku-5'      TATTGTAATTATAATTATTATTATTATTGTTAACCTTAATTATTTGACCATGATTATAAT -1429
bvorf21-5'      TATTGTAATTATAATTATTATTATTATTGTTAACCTTAATTATTTGACCATGATTATAAT -1429
                ************************************************************

fukkoku-5'      ATTATTTAATAGCAATATGAATAATCAAATAATAGACAATAATACAAGTATAATACTACA -1369
bvorf21-5'      ATTATTTAATAGCAATATGAATAATCAAATAATAGACAATAATACAAGTATAATACTACA -1369
                ************************************************************

fukkoku-5'      CATTGTGGTACTTTAATAAAAAATTCTAATAATAACATAATCAGCTAATAGTAATATGAA -1309
bvorf21-5'      CATTGTGGTACTTTAATAAAAAATTCTAATAATAACATAATCAGCTAATAGTAATATGAA -1309
                ************************************************************

fukkoku-5'      TAATAAAATAATAGACATAATGCAGATAAATAACAAAATAATAGACATAATACAAATAAA -1249
bvorf21-5'      TAATAAAATAATAGACATAATGCAGATAAATAACAAAATAATAGACATAATACAAATAAA -1249
                ************************************************************

fukkoku-5'      CAATAAAGTAATAGACATTAATACAAGTATAATATTATATAATCATTGTGGTACTTTAAT -1189
bvorf21-5'      CAATAAAGTAATAGACATTAATACAAGTATAATATTATATAATCATTGTGGTACTTTAAT -1189
                ************************************************************

fukkoku-5'      TAAAATTCTAATAATAACATAATCAACTAATAGTGATATGAAATTATGAATAACAAAATA -1129
bvorf21-5'      TAAAATTCTAATAATAACATAATCAACTAATAGTGATATGAAATTATGAATAACAAAATA -1129
                ************************************************************

fukkoku-5'      ATGGACAATAATACAAATGTATATTAAACATTGACTATTTGGACCTTATTGGACCTTATT -1069
bvorf21-5'      ATGGACAATAATACAAATGTATATTAAACATTGACTATTTGGACCTTATTGGACCTTATT -1069
                ************************************************************

fukkoku-5'      AAACCTGATTGAAACTTATTGGACCTTATTAGACCTGATTGGAACTTATTGCACCTAATT -1009
bvorf21-5'      AAACCTGATTGAAACTTATTGGACCTTATTAGACCTGATTGGAACTTATTGCACCTGATT -1009
                ******************************************************** ***

fukkoku-5'      GAAACTTATTGCACCTGGAACTTATTGGACCTTATTAGACCTTATTGGAAGTTATTGCCC -949
bvorf21-5'      GAAACTTATTGCACCTGGAACTTATTGGACCTTATTAGACCTTATTGGAAGTTATTGCCC -949
                ************************************************************

fukkoku-5'      TTATTAGACCTTATTACAACTTATCTGAACTTATTGGACCTGAAACTTAATTTTTTTAAG -889
bvorf21-5'      TTATTAGACCTTATTACAACTTATCTGAACTTATTGGACCTGAAACTTAATTTTTTTAAG -889
                ************************************************************

fukkoku-5'      TTGAGCAGAACGCACCCTAAATCTCCAATTATAAAATAACATAGGTCGGAATAAAAAGAA -829
bvorf21-5'      TTGAGCAGAACGCACCCTAAATCTCCAATTATAAAATAACATAGGTCGGAATAAAAAGAA -829
                ************************************************************

fukkoku-5'      AGTATTGTAAATGAACATTTAGTCTAAATTTAAAGAACCTAACTTTTAAATGTAAATTTG -769
bvorf21-5'      AGTATTGTAAATGAACATTTAGTCTAAATTTAAAGAACCTAACTTTTAAATGTAAATTTG -769
                ************************************************************

fukkoku-5'      AAAAAAGGAATCTCGCCAACAACCATTTTTCCTAAAAGGTAAAACAAGTTGCAAGATTTA -709
bvorf21-5'      AAAAAAGGAATCTCGCCAACAACCATTTTTCCTAAAAGGTAAAACAAGTTGCAAGATTTA -709
                ************************************************************

fukkoku-5'      ATGAAACAGATAACAACATTTTTCTTAAATCATAAATTCTTAAAATATTAAACCTATATC -649
bvorf21-5'      ATGAAACAGATAACAACATTTTTCTTAAATCATAAATTCTTAAAATATTAAACCTATATC -649
                ************************************************************

fukkoku-5'      GTTTAACAGAGGGTGCACCGTCCTTTATGCGTACTTGGATGCATGGTCCTATTTGCGCTT -589
bvorf21-5'      GTTTAACAGAGGGTGCACCGTCCTTTATGCGTACTTGGATGCATGGTCCTATTTGCGCTT -589
                ************************************************************
```

```
fukkoku-5'     TTCCATTCCCTAAAAAACCGAGTCTAAAGCTATTGTAATAAAACACTCTAGTAGTCCTCT -529
bvorf21-5'     TTCCATTCCCTAAAAAACCGAGTCTAAAGCTATTGTAATAAAACACTCTAGTAGTCCTCT -529
               ************************************************************

fukkoku-5'     CAAAAAAAAAAAA-TAATACACTCTAGTTGTCGTTGACTCTCAGCATGTGTCATTTAGAG -469
bvorf21-5'     CAAAAAAAAAAAAATAATACACTCTAGTTGTCGTTGACTCTCAGCATGTGTCATTTAGAG -469
               ************* **********************************************

fukkoku-5'     ACTCGTAACGCATTGACACACTTACTCGGGGTAGAAATATTTTGTTCCATTTATTAGAGA -409
bvorf21-5'     ACTCGTAACGCATTGACACACTTACTCGGGGTAGAAATATTTTGTTCCATTTATTAGAGA -409
               ************************************************************

fukkoku-5'     AAATTTCATCCCCTGATCTCAATTATCAAATCAACATCTAAAAATTTAAAATGACTAGGT -349
bvorf21-5'     AAATTTCATCCCCTGATCTCAATTATCAAATCAACATCTAAAAATTTAAAATGACTAGGT -349
               ************************************************************

fukkoku-5'     ACGTAACGAAAAACGAATGACTCTCGATAATAGTACACCCCATTAATCCATTCTTATTTC -289
bvorf21-5'     ACGTAACGAAAAACGAATGACTCTCGATAATAGTACACCCCATTAATCCATTCTTATTTC -289
               ************************************************************

fukkoku-5'     TTAGTTTGTTGCATAGTTTGTTGCGTAGTGCATAGCTGGCTGCAAAAGAAATCTTTTGCA -229
bvorf21-5'     TTAGTTTGTTGCATAGTTTGTTGCGTAGTGCATAGCTGGCTGCAAAAGAAATCTTTTGCA -229
               ************************************************************

fukkoku-5'     CAGAGAAAACTTTTGCACTTTCGGAATTCAGTAGGAATATCATAACCATTTATGGAAGCA -169
bvorf21-5'     CAGAGAAAACTTTTGCACTTTCGGAATTCAGTAGGAATATCATAACCATTTATGGAAGCA -169
               ************************************************************

fukkoku-5'     ACAACTCTTGTGACCCATTTCATCTAAAATCTTAATCTCGTAAATTTTACCTTTCAGAAT -109
bvorf21-5'     ACAACTCTTGTGACCCATTTCATCTAAAATCTTAATCTCGTAAATTTTACCTTTCAGAAT -109
               ************************************************************

fukkoku-5'     TCAAAAATCACATAATTTTTTTTGGTATGTTACTTGAACCCAGTTCATAACTGACCCTGA -49
bvorf21-5'     TCAAAAATCACATAATTTTTTTTGGTATGTTACTTGAACCCAGTTCATAACTGACCCTGA -49
               ************************************************************

fukkoku-5'     AATTCAAGAATTTGGAGCAAAGTTAGCAGCTTTTGTTGTTCAAAAATC -1
bvorf21-5'     AATTCAAGAATTTGGAGCAAAGTTAGCAGCTTTTGTTGTTCAAAAATC -1
               ************************************************
```

S2 Fig. Alignment of nucleotide sequences between *orf20*$_{fukkoku}$ (fukkoku-5') and *orf21* (bvorf21-5'). 5' upstream regions are shown. Nucleotides are numbered from the initiation codon. Asterisks denote matched residues. Dashes indicate deletion.
