# Supplementary material for: A fertility-restoring genotype of beet (Beta vulgaris L.) is composed of a weak restorer-of-fertility gene and a modifier gene tightly linked to the Rf1 locus
Source: PLoS One. 2018 Jun 1;13(6):e0198409. doi: 10.1371/journal.pone.0198409 (PMC5983528; doi:10.1371/journal.pone.0198409)
Supplement: S3 Fig — (PDF) [file pone.0198409.s003.pdf]

```
fukkoku-3'      CCATTTACCAACCAGCATCTTCTTTTAGCAGCTTCGCCTGTTTATGAATTATGGTAATCA 1920
bvorf18-3'      CCATTTACCAACCAGCATCTTCTTTTAGCAGCTTCGCCTGTTTATGAATTATGGTAATCA 1926
                ************************************************************

fukkoku-3'      AAATTAAACAGCTCATGGATCATTATATTGTCGTTATATTTCGTCTGTTTGACAAAGTTT 1980
bvorf18-3'      AAATTAAACAGCTCATGGATCATTATATTGTCGTTATATTTCGTCTGTTTGACAAAGTTT 1986
                ************************************************************

fukkoku-3'      AGAGGTTAATTTGAGTTGAAAACTTGCTATTAGTATGTTATCCTATCCCACTGACCTTGT 2040
bvorf18-3'      AGAGGTTAATTTGAGTTGAAAACTTGCTATTAGTATGTTATCCTATCCCACTGACCTTGT 2046
                ************************************************************

fukkoku-3'      TAATTCAATCTAATTTAGTTCCTATGTTAATTCACTGCTCTTTTTCTCCTTTAATAGATC 2100
bvorf18-3'      TAATTCAATCTAATTTAGTTCCTATGTTAATTCACTGCTCTTTTTCTCCTTTAATAGATC 2106
                ************************************************************

fukkoku-3'      CTCTCTATCCGTTTTTCTTTTTTCTTTTTGAGAAAAGGAGCATCATTGATAAATATAAAT 2160
bvorf18-3'      CTCTCTATCCGTTTTTCTTTTTTCTTTTTGAGAAAAGGAGCATCATAGATAAATATAAAT 2166
                *********************************************** ************

fukkoku-3'      CGAAGTCATTACAAACTAAACTAGAGCTTCCAATATAAGTCCCTGGAAGTCCGAAAGCAA 2220
bvorf18-3'      CGAAGTCATTACAAACTAAACTAGAGCTTCCAATATAAGTCCCTGGAAGTCCGAAAGCAA 2226
                ************************************************************

fukkoku-3'      TAGTAGTAGATAGCATAGTAGAGATGAGTTTCTCTTTTAGGTAGATAGTAGATAGGAGGA 2280
bvorf18-3'      TAGTAGTAGATAGCATAGTAGAGATGAGTTTCTCTTTTAGGTAGATAGTAGATAGGAGGA 2286
                ************************************************************

fukkoku-3'      TCGATAGATGACCCACCATTGTCGCCGCTTTCGGCGCCACCAACACCAGGGGAAGAAGAG 2340
bvorf18-3'      TCGATAGATGACCCACCAT--TTGCCGCTTTCGGCGCCACCAACACCAGGGGAAGAAGAG 2344
                *******************  * *************************************

fukkoku-3'      CCTCCAATCTGGCCACCACCGGATATAGGAATATCAATTCACTCCTTAACGTCCATGGAT 2400
bvorf18-3'      CCTCCAATCTGGCCACCACCGGATATAGGAATATCAATTCACTCCTTAACGTCCATGGAT 2404
                ************************************************************

fukkoku-3'      GTGATTACCCCATGCATGGATAAACGAGACTGTGATTGGCCCTGCATGTTTTGTTGTGGC 2460
bvorf18-3'      GTGATTACCCCATGCATGGATAAACGAGACTGTGATTGGCCCTGCATGTTTTGTTGTGGC 2464
                ************************************************************

fukkoku-3'      AAATGCAATGGCACTTGTTGCCTCAGTAAAATGATTTATGAGTAGACACATGAGATATCT 2520
bvorf18-3'      AAATGCAATGGCACTTGTTGCCTCAGTAAAATGATTTATGAGTAGACACATGAGATATCT 2524
                ************************************************************

fukkoku-3'      TCCCTTATATTATTGTTCATTTTGAGTTAGTTATACTCCCTCCTCTTTTTTCTTAGTTGC 2580
bvorf18-3'      TCCCTTATATTATTGTTCATTTTGAGTTAGTTATACTCCCTCCTCTTTTTTCTTAGTTGC 2584
                ************************************************************

fukkoku-3'      TATATTCCATTTTTGGATACAAAATCACATGAGAATTTTGACTTTCTTTAATTTTATATA 2640
bvorf18-3'      TATATTCCATTTTTGGATACAAAATCACATGAGAATTTTGACTTTCTTTAATTTTATATA 2644
                ************************************************************

fukkoku-3'      TGTAAGAAAAAAAAACATAGTATTTTATTAGATTTCTCTCAAATGTGTAATTTTCATATA 2700
bvorf18-3'      TGTAAGAAAAAAAA-CATAGTATTTTATTAGATTTCTCTCAAATGTGTAATTTTCATATA 2703
                ************** *********************************************

fukkoku-3'      TAGTTTTTTT-ATAATTTTCTCATATACATAACTCAACATATTAAGGTTTGAAGTCATGA 2759
bvorf18-3'      TAGTTTTTTTTATAATTTTCTCGTATACATAACTCAACATATTAAGGTTTGAAGTCATGA 2763
                ********** *********** *************************************
```

```
fukkoku-3'      CTGCGAGCAAGAAACCACGCAGGTGGGGCTCACAGCAGCGCCAGCAGGAAGGACGAGTGA 2819
bvorf18-3'      CTGCGAGCAAGAAACCACGCGGGTGGGGCTCACAGCAGCGCCAGCAGGAAGGACGAGTGA 2823
                ******************** ***************************************

fukkoku-3'      AAAAAATTAGGGTGAATTAGTGTGGCTCTGATACCATGACAAATTGATTAGAGCTTAATG 2879
bvorf18-3'      AAAAAATTAGGGTGAATTAGTGTGGCTCTGATACCATGACAAATTGATTAGAGTTTAATG 2883
                ***************************************************** ******

fukkoku-3'      ATTTGTGTTACTATTGTTAAACTTAGAGTTTATATATATCCTAAGTTTACATCCAAGGCC 2939
bvorf18-3'      ATTTGTGTTACTATTGTTAAACTTAGAGTTTATATATATCCTAAGTTTACATCCNAG--C 2914
                ****************************************************** **  *

fukkoku-3'      TTAGGCCCAATATATACAACTCAACATATATACAAGGTCCAATATGTATCAACAAAATGA 2999
bvorf18-3'      TTAG-CCCAATATATACAACTCAACATATATACAAGGTCCAATATGTATCAACAAAATGA 3000
                **** *******************************************************

fukkoku-3'      AAAATAAAAACATAATGTAGCAACTAGCAACTAAAAAAAGACGGAGAGAGTATTTAAGTT 3059
bvorf18-3'      AAAATAAAAACATAATGTAGCAACTAGCAACTAAAAAAAGACGGAGAGAGTATTTAATTT 3060
                ********************************************************* **

fukkoku-3'      AACAATATAATGATAATTAAATCAGTGTAAGTATTAGTCAAGATCTAGATTGAAATTACT 3119
bvorf18-3'      AACAATATAATGATAATTAAATCAGTGTAAGTATTAGTCAAGATCTAGATTGAAATTACT 3120
                ************************************************************

fukkoku-3'      CTCCGACGACCTCCCCGACCCATTTGCCTTTATGTAATCAATCATTATATACAACCTCCG 3179
bvorf18-3'      CTCCGACGACCTCCCCGACCCATTTGCCTTTATGTAATCAATCATTATATACAACCTCCG 3180
                ************************************************************

fukkoku-3'      ATTCTTTTGATGGCAACTATTTGAATGTTACCTTTATCAACATGAAACTTTAACTATTAA 3239
bvorf18-3'      ATTCTTTTGATGGCAACTATTTGAATGTTACCTTTATCAACATGAAACTTTAACTATTAA 3240
                ************************************************************

fukkoku-3'      TATCTTTATTAATTATTAACATAATTTACTACTAATTATAAATTATTTAAATATATACCT 3299
bvorf18-3'      TATCTTTATTAATTATTAACATAATTTACTACTAATTATAAATTATTTAAATATATACCT 3300
                ************************************************************

fukkoku-3'      TGAGATTAATTTTCCTAACATTTATTTACACTCTAACATTTGCTTTTATATTTTATAAAG 3359
bvorf18-3'      TGAGATTAATTTTCCTAACATTTATTTACACTCTAACATTTGCTTTTATATTTTATAAAG 3360
                ************************************************************

fukkoku-3'      AAAATGGTGATGGTTAGAAGTATATATATATGAGTAGTTCAATAGTCAAAGCCTTGCAAA 3419
bvorf18-3'      AAAATGGTGATGGTTAGAAGTATATATATATGAGTAGTTCAATAGTCAAAGCCTTGCAAA 3420
                ************************************************************

fukkoku-3'      TTAAAGAAACGGCGGAAGAGTATATTGAAAATTTATATTGTCACCTTTTTTAATAGATGA 3479
bvorf18-3'      TTAAAGAAACGGCGGAAGAGTATATTGAAAATTTATATTGTCACCTTTTTTAATAGATGA 3480
                ************************************************************

fukkoku-3'      GTCGATGATGGTTTAAAATTGAGGCTTCTAATATGCGCTAGCTTAGATTAAAGAGAACAG 3539
bvorf18-3'      GTCGATGATGGTCTAAAATTGAGGCTTCTAATATGCGCTAGCTTAGATTAAAGAGAACAG 3540
                ************ ***********************************************

fukkoku-3'      GAAATTGTTATGACAATTTGCGTAAGTTGACTGAGATCATCATGAAGTCGAGTATTAGCA 3599
bvorf18-3'      GAAATTGTTATGACAATTTGCGTAAGTTAACTGAGATCATCATGAGGTCGAGTATTAGCA 3600
                **************************** **************** **************

fukkoku-3'      ACCTTAGTAATTTATAATTCCTCCGTTTCTTTTTAATTTACTCATTTTATTTTGGGCGAG 3659
bvorf18-3'      ACCTTAGTAATTTATAATTCCTCCATTTCTTTTTAATTTACTCACTTTATTTTGGGCGAG 3660
                ************************ ******************* ***************
```

```
fukkoku-3'     AATTAAGGAAGAAGTACAAAAGTATGAGACAAAAACTGAAAAGATAAAGAGAAAAACTGA 3719
bvorf18-3'     AATCAAGGAAGAAGTACAAAAGTATGAGACAAAAACTGAAAAGATAAAGAGAAAAACTGA 3720
               *** ********************************************************

fukkoku-3'     AAAGTGCGAGAAATAAAGTAAAAAAGTGGGTTAAAAATATTAAAAAGTGAATCACATGGG 3779
bvorf18-3'     AAAGTGCGAGAAATAAAGTAAAAAAGTGGGTTAAAATTATTAAAAAGTGGATCACTTGGG 3780
               ************************************ ************ ***** ****

fukkoku-3'     TAGAAAAGGAGGAGAAAATAGATGTGACATGTTACCAAAAATAGAAAAAGCAGCAGTGTC 3839
bvorf18-3'     TAGAAAAGGAGGAGAAAATAGATGTGACATGTTACCAAAAATAGAAAAAGCAGCACTGTC 3840
               ******************************************************* ****

fukkoku-3'     ACAGTGTGCAGATTAAAAAGAAACAAACCAAAAAAAAATGTATAAATTAAAAAGAAATGG 3899
bvorf18-3'     ACAGTGTGCAGATTAAAATGAAACAAACCAAAAAAAAATATATAAATTAAAAAGAAATGG 3900
               ****************** ******************** ********************

fukkoku-3'     AGGGAGTAATACTTAACACATAAATATTGTTTATTAATTGAAGCTCGAGCTTCACTTAGA 3959
bvorf18-3'     AGGGAGTAATACTTAACACATAAATATTGTTTACTAATTGAAGCTCGAGCTTCACTCAGA 3960
               ********************************* ********************** ***

fukkoku-3'     TACATGATTTTTAATTTTTTATGACCTATCATAGTAAGTTTGCGGTGCTACTTTGATCCT 4019
bvorf18-3'     TACATGATTTTTAATTTTTTATGACCTATCATAGTAAGTTTGCGGTGCTACTTTGATCCT 4020
               ************************************************************

fukkoku-3'     TCCTTTATGATAAGACACATCCCTTTATTCTCTAAGAGAATCACTTAAATTTATAAGGTA 4079
bvorf18-3'     TCCTTTATGATAAGACACATCCCTTTATTCTCTAAGAGAATCACTTAAATTTATAAGATA 4080
               ********************************************************* **

fukkoku-3'     TAAAGTTAACGCTATAAGTTAAAGAGGTCATTTTTAGATAATAGGTCACCTTGTATGTAA 4139
bvorf18-3'     TAAAGTTAACGCTATAAGTTAAAGAGGTCATTTTTAGATAATAGGTCACCTTGTATGTAA 4140
               ************************************************************

fukkoku-3'     ATAGGTCACTTCTACTAAAAATTTTCCCGTATAGCAAGTTACAATTGTTTTAACCCAAAT 4199
bvorf18-3'     ATAGGTCACTTCTACTAAAATTTTTCCCGTATAGCAAGTTACAATTGTGTTAACCCAAAT 4200
               ******************** *************************** ***********

fukkoku-3'     TATGGTAACATTTTGTACTTTTATCAACTCCGACTCATACTTGATATTCGAATAACCTCA 4259
bvorf18-3'     TATGGTAACATTTTGTACTTTTATCAACTCCGACTCATACTTGATATTCGAATAACCTCA 4260
               ************************************************************

fukkoku-3'     CTTATCAAGTCAATGCAAGCAAATCATGTTAAACTTTTGTTCGCCTTAATGTCGAGACCA 4319
bvorf18-3'     CTTATCAAGTCAATGCAAGCAAATCATGTTAAACTTTTGTTCGCCTTAATGTCGAGACCA 4320
               ************************************************************

fukkoku-3'     GTCTATCTAAAAGAACTTTTTTTTTTTT-ACAATTACCTAAAAGAACTTATTATTAGTTA 4378
bvorf18-3'     GTCTATCTAAAAGAACTTTTTTTTTTTTTACAATTACCTAAAAGAACTTATTATTAGTTA 4380
               **************************** *******************************

fukkoku-3'     TTGGCTACAAAACCACACAAATATTCAATACAATCTCAAGCTACAACTTCAAAGATTAAA 4438
bvorf18-3'     TTGGCTACAAAACCACACAAATATTCAATACAATCTCAAGCTACAACTTCAAAGATTAAA 4440
               ************************************************************

fukkoku-3'     GATTCCCTCACCCATCACATTCATATACAATATATTGGTCTATCAGTGATGTGATTGAAT 4498
bvorf18-3'     GATTCCCTCACCCATCACATTCATATACAATATATTGGTCTATCAGTGATATGATTGAAT 4500
               ************************************************** *********

fukkoku-3'     AAGTTGCACTCAATTGTATTGACCTACATAAGTATGGCCTTTATAATTACATAAAGGTGA 4558
bvorf18-3'     AAGTTGCACTCAATTGTATTGACCTACATAAGTATGGCCTTTATAATTACATAAAGGTGA 4560
               ************************************************************
```

```
fukkoku-3'      TCCAAAGTTCCAATAATATCAATGAGATTTGAAGATTGTTTCCAGATATTCACTCAACCA 4618
bvorf18-3'      TCCAAAGTTCCAATAATATCAATGAGATTTGAAGATTGTTTCCAGATATTCACTCAACCA 4620
                ************************************************************

fukkoku-3'      AATATACCTTACGACAACTTCATCCTGAAATCTTCGGTTTGTGACAGTACTCCACTTTTA 4678
bvorf18-3'      AATATACCTTACGACAACTTCATCCTGAAATCTTCGGTTTGTGACAGTACTCCACTTTTA 4680
                ************************************************************

fukkoku-3'      ATTCAATGGTCAAATATTTATACCTTAACCCTTAATTCAATGCCCATAATGTTGATGATC 4738
bvorf18-3'      ATTCAATGGTCAAATATTTATACCTTAACCCTTAATTCAATGCCCATAATGTTGATGATC 4740
                ************************************************************

fukkoku-3'      ATGTGAAGTAGTGCAATATATAGCTTTACTTCATCTAAAAATGCATTAAAAAATTTAGTC 4798
bvorf18-3'      ATGTGAAGTAGTGCAATATATAGCTTTACTTCATCTAAAAATGCATTAAAAAATTTAGTC 4800
                ************************************************************

fukkoku-3'      AATTGATTGAATATAAACTAGCTAACTCGTGTACTACTCAACCAAGATGGTTTCATGTCA 4858
bvorf18-3'      AATTGATTGAATATAAACTAGCTAACTCGTGTACTACTCAACCAAGATGGTTTCATGTCA 4860
                ************************************************************

fukkoku-3'      ATGATAAATAGCTCATTGTTTCTGAACCATATCATCTCCTTCATCACACCATGAAGGGAG 4918
bvorf18-3'      ATGATAAATAGCTCATTGTTTCTGAACCATATCATCTCCTTCATCACACCATGAAGGGAG 4920
                ************************************************************

fukkoku-3'      CTTCTTTCAAGCTGTCGATTCTGATTTTCGTCCTTGTGGCTTTCGCTTCGTGTAAGTGTC 4978
bvorf18-3'      CTTCTTTCAAGCTGTCGATTCTGATTTTCGTCCTTGTGG-TTTCGCTTCGTGTAAGTGTC 4979
                *************************************** ********************

fukkoku-3'      TTTTATCTCTTATGATTCTTGCATGTAAATTAAGATGCATACATGGTTTGTCTTGATATA 5038
bvorf18-3'      TTTTATCTCTTATGATTCTTGCATGTAAATTAAGATGCATACATGGTTTGTCTTGATATA 5039
                ************************************************************

fukkoku-3'      GTTTTCTTCACCTAAATAGCTCTTCTAGTATTATTATTGATTGGTTGGGGTCATACTATA 5098
bvorf18-3'      GTTTTCTTCACCTAAATAGCTCTTCTAGTATTATTATTGATTGGTTGGGGTCATACTATA 5099
                ************************************************************

fukkoku-3'      TTGCAGTTCTTTGTCCATTTACAGCAGAAGCAAGACACAAGCACGTGCATCATAGTAATT 5158
bvorf18-3'      TTGCAGTTCTTTGTCCATTTACAGCAGAAGCAAGACACAAGCACGTGCATCATAGTAATT 5159
                ************************************************************

fukkoku-3'      GCCACCATCGTCGTGGACGTCCTGATCCAGCACCAACACCGGCGCCTGTGCCAGCATCTA 5218
bvorf18-3'      GCCACCATCGTCGTGGACGTCCTGATCCAGCACCAACACCGGCGCCTGTGCCAGCATCTA 5219
                ************************************************************

fukkoku-3'      ATGAAGGCATACCACCATATCAAATCCATGGTTGTGGTTACCCATGTAGTGACTCCAACG 5278
bvorf18-3'      ATGAAGGCATACCACCATATCAAATCCATGGTTGTGGTTACCCATGTAGTGACTCCAAAG 5279
                ********************************************************** *

fukkoku-3'      ACTGTGATTGGCCTTGTACAGAATGCGGTGTCAACAGAACTTGTGCTTATGAAGAGCCCT 5338
bvorf18-3'      ACTGTGATTGGCCTTGTACAGAATGCGGTGTCAACAGAACTTGTGCTTATGAAGAGCCCT 5339
                ************************************************************

fukkoku-3'      TCTTTCCATCACCATCACCTGTTCCATCACCAAGTATGGAAACCCCAACACAAGAACCAC 5398
bvorf18-3'      TCTTTCCATCACCATCACCTGTTCCATCACCAAGTATGGAAACCCCAACACAAGAACCAC 5399
                ************************************************************

fukkoku-3'      CACTGTCACCAGCACCGGCACCTGATAATGGCATTGGCGTAGGTGTGCCACCATATCAAA 5458
bvorf18-3'      CACTGTCACCAGCACCGGCACCTGATAATGGCATTGGCGTAGGTGTGCCACCATATCAAA 5459
                ************************************************************
```

```
fukkoku-3'      TCCACGGTTGTGGCTACCCATGTAGTGACTCCAATGATTGTGATTGGCCTTGTACAGTAT 5518
bvorf18-3'      TCCACGGTTGTGGCTACCCATGTAGTGACTCCAATGATTGTGATTGGCCTTGTACAGTAT 5519
                ************************************************************

fukkoku-3'      GTGGTGTCAACGGAACTTGTGCTTTTGAAGAGCCATTCTTTCCATCATCA---CCTGTTC 5575
bvorf18-3'      GTGATGTCAACAGAACTTGTGCTTTTGAAGAGCCCTTCTTTCCATCATCATCACCTGTTC 5579
                *** ******* ********************** ***************   *******

fukkoku-3'      CATCACCAAGTATAGAAACTCCAACACAAGAACCACCACTATCACCAGCACCGGCACCTG 5635
bvorf18-3'      CATCACCAAGTATGGAAACCCCAACACAAGAACCACCACTATCACCAGCACCGGCACCTG 5639
                ************* ***** ****************************************

fukkoku-3'      ATAATGGCATTGGCGTAGGTGTGCCACCATATCAAATCCACGGTTGTGGCTACCCATGTA 5695
bvorf18-3'      ATAATGACATTGGCGTAGGTGTGCCACCATATCAAATCCACGGTTGTGGCTACCCATGTA 5699
                ****** *****************************************************

fukkoku-3'      GTGACTCTAATGATTGTGATTGGCCTTGTACAATCTGTGGTGCTGACCAAACCTGTACTT 5755
bvorf18-3'      GTGACTCCAATGATTGTGATTGGCCTTGTACAATCTGTGGTGCTGACCAAACCTGTACTT 5759
                ******* ****************************************************

fukkoku-3'      TTGATGAACCCTTCTTTACATCACCCTCGCTTGCTCCATTACCTCTCACAGAAGCACCCA 5815
bvorf18-3'      TTGATGAGCCCTTCTTCACATCACCCTCGCCTGCTCCATTGCCTCTCACAGAAGCACCCA 5819
                ******* ******** ************* ********* *******************

fukkoku-3'      TTCCACAATGGGTACCTGGTAATGGTATCGCGGCACCACCATATCAGATTCATGGTTGTG 5875
bvorf18-3'      TTCCACAATGGGTACCTGGTAATGGCATCGCGGCACCACCATATCAGATTCATGGTTGTG 5879
                ************************* **********************************

fukkoku-3'      GTTACCCATGTAACGACTCCAATGACTGTGATGCACCCTGTACAGTCTGTTGTGCAAACT 5935
bvorf18-3'      GTTACCCATGTAACGACTCCAATGACTGTGATGCACCCTGTACAGTCTGTTGTGCAAACT 5939
                ************************************************************

fukkoku-3'      ATACCTGCTGTTATGATGTGGCTGATCCTGAGTACATGCTACCACCTATGTCACCCTCTG 5995
bvorf18-3'      ATACCTGCTGTTATGATGTGGCTGATCCTGAGTACATGCTACCACCTATGTCACCCTCTG 5999
                ************************************************************

fukkoku-3'      AGCCACCGAAATTATTACCTCTTCCACCATCTCCACCTCCATCTACAGAGGATGTAGAAA 6055
bvorf18-3'      AGCCACCGAAATTATTACCTCTTCCACCATCTCCACCTCCATCTACAGAGGATGTAGAAA 6059
                ************************************************************

fukkoku-3'      ATGATGATATGTTTGCACCACAACCTGCTTATGATATAGGAACGCCTGCGGAACTTCCAC 6115
bvorf18-3'      ATGATGATATGTTTGCACCACAACCTGCTTATGATATAGGAACGC-TGCGGAACTTCCAC 6118
                ********************************************* **************

fukkoku-3'      CACCAGGATACGAATTCCCGCCGTATCAAATCCATGGTTGTGGCTATGGCCCCTGCATGG 6175
bvorf18-3'      CACCAGGATACGAATTCCCGCCGTATCAAATCCATGGTTGTG-CTATGGCCCCTGCATGG 6177
                ****************************************** *****************

fukkoku-3'      ACTCCAACGACTGCGATTGGCCCTGCACATCCTGCTGCTCTAATCATACATGTTGCTATG 6235
bvorf18-3'      ACTCCAACGACTGCGATTGGCCCTGCACATCCTGCTGCTCTAATCATACATGTTGCTATG 6237
                ************************************************************

fukkoku-3'      AGGAGCCTATGTTTCGATGAAAATCCTCAAACACACAAATTCCAAAGAAAAAGTATAGTA 6295
bvorf18-3'      AGGAGCCTATGTTTCGATGAAAATCCTCAAACACACAAATTCCAAAGAAAAAGTATAGTA 6297
                ************************************************************

fukkoku-3'      ACACAATGTAATAAAACTTAGTGTTCCTGTATTACTTAAAATCACATTTGACCTATATTC 6355
bvorf18-3'      ACACAATGTAATAAAACTTGGTGTTCCTGTATTACTTAAAATCACATTTGACCTATATTC 6357
                ******************* ****************************************
```

```
fukkoku-3'      TTCAATTGCTATGTCATTGTCTAATGATTGAAAGCAAGTACTTTTATTTCTGTGTCATAC 6415
bvorf18-3'      TTCAATTGCTATGTCATTGTCTAATGATTGAAAGCAAGTACTTTTATTTCTGTGTCATAC 6417
                ************************************************************

fukkoku-3'      AAATGTAAGCAAGATCAATAAAGAATATATACAACTACGTTAATCAATTGCTACTATTAA 6475
bvorf18-3'      AAATGTAAGCAAGATCAATAAAGAATATATACAACTACGTTAATCAATTGCTACTATCAA 6477
                ********************************************************* **

fukkoku-3'      ACTGCATTTTCTATCAAACAACAGTATAATTATTCTGACGTACTCTAATAATTAAAGTGG 6535
bvorf18-3'      ACTGCATTTTCTATCAAACAACAGTATAATTATTCTGACGTACTCTAATAATTAAAGTGG 6537
                ************************************************************

fukkoku-3'      GTTGAAC--------TAAGCCTTTAATGACTGATTCATCTGCTCTACGCCTATATCACAA 6587
bvorf18-3'      GTTGAACAGTTGAACTAAGCCTTTAATGACTGATTCATCTGCTCTACGCCTATATCACAA 6597
                *******        *********************************************

fukkoku-3'      TTTCAGAGTAGATACATTGCGAGATTTCTGTTGTAAGAACCATTTTGATCATGTTGCTGT 6647
bvorf18-3'      TTTCAGAGTAGATACATTGCGAGATTTCCGTTGTAAGAACCATTTTGATCATGTTGCTGT 6657
                **************************** *******************************

fukkoku-3'      TACAGACAACGTGATTTTCAGTATATTTTGTTTACAAACTTCCTTCGTCGAGGAAATTCT 6707
bvorf18-3'      TACAGACAACATGATTTTCAGTATATTTTGTTTACAAATTTCCTTCGTCGAAGAAATTCT 6717
                ********** *************************** ************ ********

fukkoku-3'      GGCATGTATTATTAAGATACCAACTTAAATTCTGGTATAGGTTCCATTCTAATTAATATC 6767
bvorf18-3'      GACATGTATTATTAAGATACCAACTTGAATTCTGGTATATGTTCCATTCTAATTAATATC 6777
                * ************************ ************ ********************

fukkoku-3'      ATCGGCATACTCCTCAACTTAGATTCATTCTTAGTTGATTTCACATCTTAATTCGTCAAC 6827
bvorf18-3'      ACCGGCATACTCCTCAACTTAGATTCATTCTTAGTTGATTTCACATCTTAATTCGTCAAC 6837
                * **********************************************************

fukkoku-3'      TTTCATAGAGAACTCATCACAGGATAAGCCAGTTTAGTTCAACAATAGTATATTCCTCCG 6887
bvorf18-3'      TTTCATAGAGAACTCATCACAGGATAAGCCAGTTTAGTTCAGCAATAGTATATTCC-CCA 6896
                ***************************************** ************** ** 

fukkoku-3'      CCAAAATCCACATAATGTTTTGAGAGTTTTAATTGCAAGTGGTATACCATCATGCTTTTT 6947
bvorf18-3'      CCAAAATCCACATAATGTTTTGAGAGTTTTAATTGCAAGTG-TATACCATCATGCTTTTT 6955
                ***************************************** ******************

fukkoku-3'      CAGTTTTCACTTTCGATAAATCAA-------------AATCAATATA------------- 6981
bvorf18-3'      CAGTTTTGACTT--GATAAATTCATCCGTTTCATATTATTCGCTATACTTTTCAATTCAG 7013
                ******* ****  *******  *             * **  ****

fukkoku-3'      AATCCT------TTCCTCAAAAAGA--------------AAAAACGATATAAATCC---- 7017
bvorf18-3'      AGTCCTAGGCAATTTTTGGAAAAGAGAGAGATAGAGAGTAAAAAGAAAAGGGACCCATGT 7073
                * ****      **  *  ******              *****  * *   * **

fukkoku-3'      -------------TAGCTCCAATTCGT-------AAAAGAATTTTACCAAAGTACTTC-- 7055
bvorf18-3'      GAGAGGAAAGAGATAGAGAGAGTTTGTTGCCCAAAAAAAAAGTGTAGCAAATAATGTGAA 7133
                             ***    * ** **       **** ** * ** ****  *  *

fukkoku-3'      -CTCCGTTTCGTTTCAAATGCAACAAAAGGGTATTATTTGTGAGATATAAAATTTCCAAT 7114
bvorf18-3'      ACTTCCCATAATGAAAAGTGTAGCGA----GTATTAT--GAAACGGATGAAGTA---AAT 7184
                 ** *   *  *   ** ** * * *    *******  *  *   ** ** *    ***

fukkoku-3'      TGTTGCGTTTAAAACGAGATGGAG----------------------GAAGCATTAAAAGT 7152
bvorf18-3'      CCTTGC-CTCAAAAAGAAAAAGCGTTATAAATCCTAGCTCCAATTCGAAGTATTAAAAAT 7243
                  ****  * **** ** *  * *                      **** ******* *
```

```
fukkoku-3'      TGTAAGACAACAAAATGAAAACAAATGGATAAATTTCATAATATATTCAACTCTACCTTC 7212
bvorf18-3'      TGTAAGACAGCAAAATGAAAACAAATGGATAAATTTCATAATATATTCAACTCTACCTTC 7303
                ********* **************************************************

fukkoku-3'      TTTTGCTCAACATCACATACACACATCCGCACCTGACAATCATTATTCATTAAAACACCC 7272
bvorf18-3'      TTTTGCTCAACATCACATGAACACATCCGCACCTGACAATCATTCTTCATTAAAACACCC 7363
                ******************  ************************ ***************

fukkoku-3'      GATAAAATAAATACCAGTTCTGAAATCATGGATATGAAACTCACAGTAAAAAGAGTATA- 7331
bvorf18-3'      AATAAAATAAATACCAGTTCTGAAATCATGGATATGAAACTCACAGTAAAAAGAGTATAG 7423
                 **********************************************************

fukkoku-3'      AAAAACACCAGCTGAAGCAAACACAAGATCAACAAAGAGGTAAAAATCAAAAAATGGTCG 7391
bvorf18-3'      AAAAACACCAGCTGAAGAAAACACAAGATCAACAAAGAGGTAAAAATTAAAAAATGGTCG 7483
                ***************** ***************************** ************

fukkoku-3'      TATCAAAATATTATTCTCTACTTCTTGTCTTCTTTCTCGGCTTCAGCAGCCCTCTTTAAT 7451
bvorf18-3'      TATCAAGATATTATTCTCTACTTCTTATCTTCTTTCTCGGCTTCAGCAGCCCTCTTTAAT 7543
                ****** ******************* *********************************

fukkoku-3'      C 7452
bvorf18-3'      C 7544
                *
```

S3 Fig. Alignment of nucleotide sequences between *orf20fukkoku* (fukkoku-3') and *orf18* (bvorf18-3'). Downstream regions are shown. Nucleotides are numbered from the initiation codon. Asterisks denote matched residues. Dashes indicate deletion.
