# Supplementary figures and images for: A fertility-restoring genotype of beet (Beta vulgaris L.) is composed of a weak restorer-of-fertility gene and a modifier gene tightly linked to the Rf1 locus
Source: PLoS One. 2018 Jun 1;13(6):e0198409. doi: 10.1371/journal.pone.0198409 (PMC5983528; doi:10.1371/journal.pone.0198409)

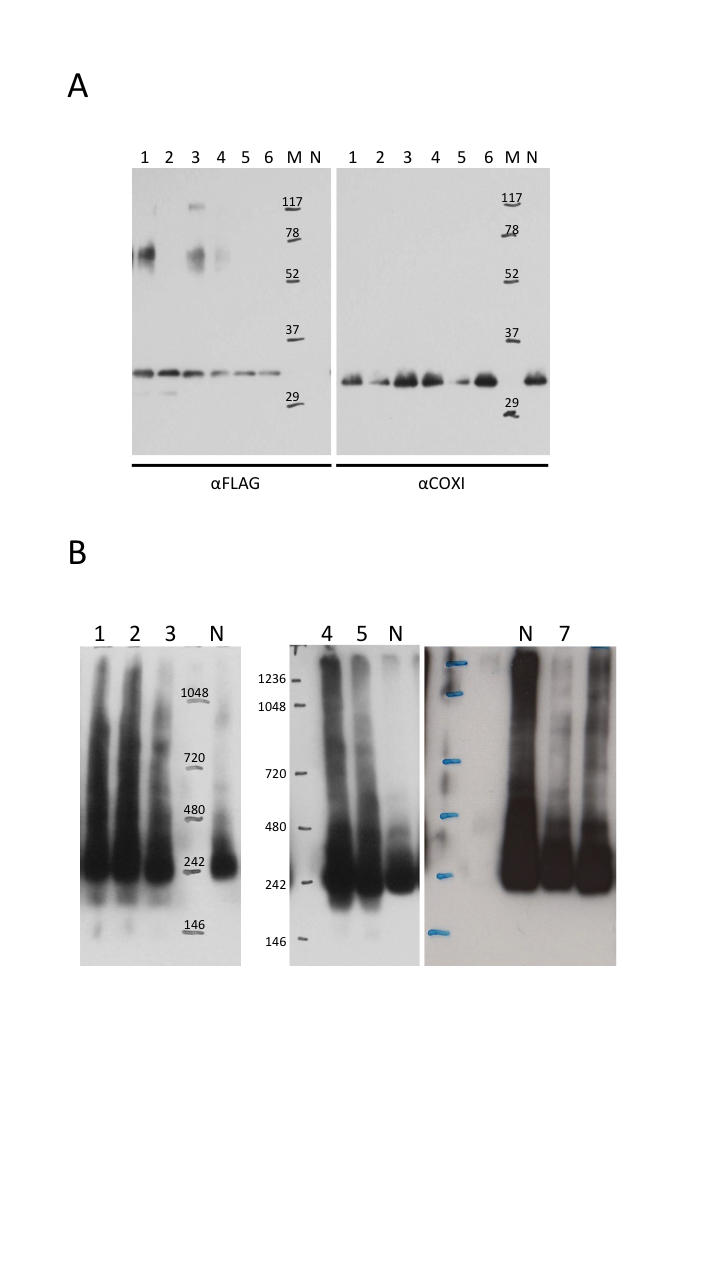

Supplement: S1 File — (TIF) [file pone.0198409.s004.tif]

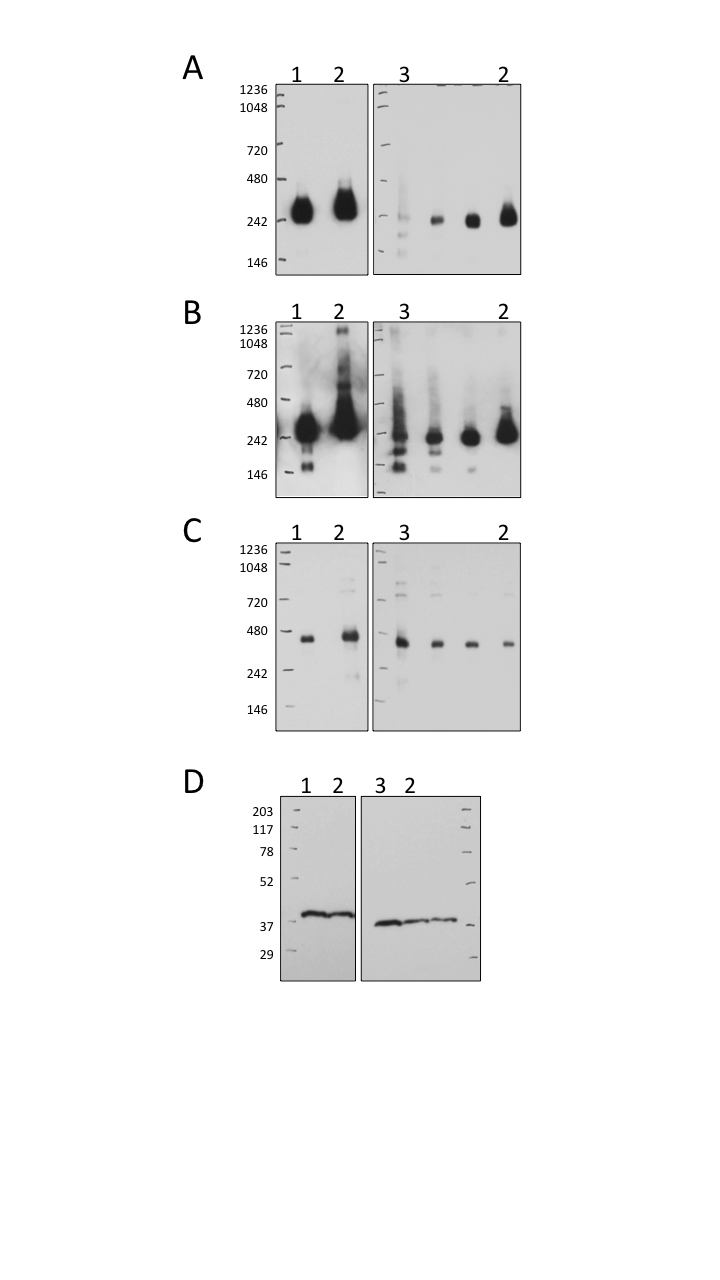

Supplement: S2 File — (TIF) [file pone.0198409.s005.tif]
