# Supplementary material for: A fertility-restoring genotype of beet (Beta vulgaris L.) is composed of a weak restorer-of-fertility gene and a modifier gene tightly linked to the Rf1 locus
Source: PLoS One. 2018 Jun 1;13(6):e0198409. doi: 10.1371/journal.pone.0198409 (PMC5983528; doi:10.1371/journal.pone.0198409)
Supplement: S1 Text — (PDF) [file pone.0198409.s006.pdf]

Segregation and phenotypic data in the F2 population derived from TA-33BB-CMS x 'Fukkoku-ouba'

We selected a plant (ID 14-76) from one of the seven fertility-restored plants of TA-33BB-CMS x 'Fukkoku-ouba' #2 (see text, ***Restorer-or-fertility*** **gene in 'Fukkoku-ouba'**). The 14-76 was selfed to obtain the F2 population. Segregation of o7 and s17 was examined as mentioned in the text (**Genotyping by DNA markers** in **Materials and methods**). Honma et al. (2014) reported a cleaved amplified polymorphic sequence marker that was linked to *Rf2*. Segregation of this marker, ca4, was examined as follows: nucleotide sequences of primers are 5'-GAGAACATGAAATTGCTGCCTG-3' and 5'-GTCCACCGGAAGAATGACC-3'; HindIII was used to detect polymorphism. Male fertility was evaluated as described in **Materials and methods**. The obtained data are shown in Table S1.

S1 Table. Types of six DNA markers and male-fertility indices of F2 plants derived from TA-33BB-CMS x 'Fukkoku-ouba' 14-76.

	Chromosome 4[1]		Chromosome 3[1]	
Plant ID	ca4	o7	s17	Male-fertility index
TZ01	FF	FF	FT	1.2
TZ02	FF	FF	TT	0.67
TZ03	FF	FF	TT	1
TZ04	FT	FT	FT	2
TZ05	TT	TT	TT	0
TZ06	FT	FF	FT	1
TZ07	TT	TT	FT	0
TZ08	FF	FF	FT	2.2
TZ09	FF	FF	TT	0.2
TZ11	FT	FT	FF	2
TZ12	FT	FT	FT	2.17
TZ13	FT	FT	FT	1.6
TZ14	FT	TT	TT	0
TZ15	TT	TT	FT	0.57
TZ16	FT	FF	FF	1.6

TZ17	FT	FT	FT	2.33
TZ18	FT	FT	FT	0.8
TZ19	TT	TT	FF	2.2
TZ20	FT	FT	FT	2
TZ21	TT	TT	TT	0
TZ22	TT	TT	FT	0.67
TZ23	FF	FF	FT	1
TZ24	FT	FT	FT	1.6
TZ25	FT	FT	TT	1.57
TZ26	TT	TT	FT	0.43
TZ27	TT	TT	FF	0
TZ28	FT	FT	FT	2.4
TZ29	FT	FT	FT	2
TZ30	FT	FT	FT	1.8
TZ31	FT	FT	FT	1
TZ32	FT	FT	FF	1.88
TZ33	FT	FT	FT	1.8
TZ34	FF	FF	FT	1.5
TZ35	FT	FT	FT	2.2
TZ36	FT	FT	FT	1.57
TZ37	FF	FT	FF	2
TZ38	FT	FT	TT	0.6
TZ39	FT	FT	FT	2
TZ40	TT	TT	TT	0
TZ41	TT	TT	FT	0
TZ42	TT	TT	TT	0
TZ43	FT	FT	FT	1.8
TZ44	TT	TT	FT	0
TZ45	TT	TT	FF	0
TZ46	TT	TT	FT	0
TZ47	TT	TT	FT	0
TZ48	FF	FF	FT	1

TZ49	FT	FT	FT	1.67
TZ50	FT	FT	TT	0
TZ51	FT	FT	FT	2
TZ52	TT	TT	FF	0
TZ53	TT	TT	FT	0
TZ54	FF	FF	FT	1.8
TZ55	TT	TT	FT	0.43
TZ56	TT	TT	FF	0
TZ57	FT	FT	FF	1.29
TZ58	FT	FT	TT	0
TZ59	TT	TT	FT	0
TZ60	FT	FT	FF	1.8
TZ61	TT	TT	TT	0
TZ62	FF	FF	TT	0.8
TZ63	FF	FT	FF	2
TZ64	FF	FF	FF	1.86
TZ65	FT	FT	FT	1.6
TZ66	FT	FT	FT	2
TZ68	TT	TT	FF	0
TZ69	FT	FT	FF	1.6
TZ70	FT	FT	FT	2.17
TZ71	FT	FT	FF	2.2
TZ72	FT	FT	TT	1
TZ73	TT	FT	TT	0
TZ74	FT	FT	FT	1.43
TZ75	FT	FT	FT	2.2
TZ76	FT	FT	FT	1.6

[1]F, 'Fukkoku-ouba'-type allele; and T, TA-33BB-CMS-type allele.

In this F2 population, marker-types of ca4 and o7 are summarized in Table S2.

S2 Table. Marker types of ca4 and o7 and plant numbers.

		ca4[1]			Total
		FF	FT	TT	
o7[1]	FF	11	2	0	13
	FT	2	35	1	38
	TT	0	1	22	23
Total		13	38	23	74

[1]F, 'Fukkoku-ouba'-type allele; and T, TA-33BB-CMS-type allele.

We tested the null hypothesis that the observed segregation of ca4 was deviated from the expected ratio, and failed to reject (p=0.263; Fisher's exact test). Linkage between ca4 and o7 was apparent, which was statistically supported (p=4.49 x 10^{-23}; Fisher's exact test). From the Table S2, the map distance between ca4 and o7 was calculated as 4 cM by using the Kosambi Function.

Reference

Honma Y, Taguchi K, Hiyama H, Yui-Kurino R, Mikami T, Kubo T, Molecular mapping of *restorer-of-fertility 2* gene identified from a sugar beet (*Beta vulgaris* L. ssp. *vulgaris*) homozygous for the non-restoring *restorer-of-fertility 1* allele, Theoretical and Applied Genetics, 127: 2567-2574, 2014.

Kosambi DD. The estimation of map distance. Ann Eugenics. 1944;12:505-525.
